# Myeloperoxidase Inhibitory and Antioxidant Activities of (*E*)-2-Hydroxy-α-aminocinnamic Acids Obtained through Microwave-Assisted Synthesis

**DOI:** 10.3390/ph14060513

**Published:** 2021-05-27

**Authors:** Astrid Rivera-Antonio, Martha Cecilia Rosales-Hernández, Irving Balbuena-Rebolledo, José Martín Santiago-Quintana, Jessica Elena Mendieta-Wejebe, José Correa-Basurto, Juan Benjamín García-Vázquez, Efrén Venancio García-Báez, Itzia I. Padilla-Martínez

**Affiliations:** 1 Laboratorio de Química Supramolecular y Nanociencias, Unidad Profesional Interdisciplinaria de Biotecnología, Instituto Politécnico Nacional, Avenida Acueducto s/n, Barrio la Laguna Ticomán, Ciudad de México 07340, Mexico; astridmriveraa@gmail.com (A.R.-A.); irving.balbu@gmail.com (I.B.-R.); martinsnqn3@gmail.com (J.M.S.-Q.); efren1003@yahoo.com.mx (E.V.G.-B.); 2 Laboratorio de Biofísica y Biocatálisis, Sección de Estudios de Posgrado e Investigación, Escuela Superior de Medicina, Instituto Politécnico Nacional, Plan de San Luis y Salvador Díaz Mirón s/n, Casco de Santo Tomas, Ciudad de México 11340, Mexico; marcrh2002@yahoo.com.mx (M.C.R.-H.); jesmenwej@yahoo.com (J.E.M.-W.); 3 Laboratorio de Diseño y Desarrollo de Nuevos Fármacos e Innovación Biotecnológica, Escuela Superior de Medicina, Instituto Politécnico Nacional, Plan de San Luis y Díaz Mirón s/n, Col. Casco de Santo Tomas, Ciudad de México 11340, Mexico; corrjose@gmail.com (J.C.-B.); benjagv_5202@hotmail.com (J.B.G.-V.)

**Keywords:** (*Z*)-2-hydroxycinnamic acid, (*E*)-dehydro-Phe, hypochlorous acid, antioxidants, 3-acetamidocoumarins, 3-aminocoumarins

## Abstract

Myeloperoxidase (MPO) is an enzyme present in human neutrophils, whose main role is to provide defenses against invading pathogens. However, highly reactive oxygen species (ROS), such as HOCl, are generated from MPO activity, leading to chronic diseases. Herein, we report the microwave-assisted synthesis of a new series of stable (*E*)-(2-hydroxy)-α-aminocinnamic acids, in good yields, which are structurally analogous to the natural products (*Z*)-2-hydroxycinnamic acids. The radical scavenging activity (RSA), MPO inhibitory activity and cytotoxicity of the reported compounds were evaluated. The hydroxy derivatives showed the most potent RSA, reducing the presence of DPPH and ABTS radicals by 77% at 0.32 mM and 100% at 0.04 mM, respectively. Their mechanism of action was modeled with BDE_OH_, IP and ΔE_H-L_ theoretical calculations at the B3LYP/6 − 31 + G(d,p) level. Compounds showed in vitro inhibitory activity of MPO with IC_50_ values comparable to indomethacin and 5-ASA, but cytotoxicities below 15% at 100–200 µM. Docking calculations revealed that they reach the amino acid residues present in the distal cavity of the MPO active site, where both the amino and carboxylic acid groups of the α-aminopropenoic acid arm are structural requirements for anchoring. (*E*)-2-hydroxy-α-aminocinnamic acids have been synthesized for the first time with a reliable method and their antioxidant properties demonstrated.

## 1. Introduction

Myeloperoxidase (MPO) is a hemeprotein member of the peroxidase family that can be found in the azurophil granules of polymorphonuclear neutrophils. The crucial role of MPO in the antimicrobial activity of neutrophils relies on the production of hypochlorous acid (HOCl) [1]. This strong oxidant species is able to cross the cell membrane, promoting not only chlorination of lipids, nucleic acids and carbohydrates but also the deamination of amino acids [2,3], which are strongly associated with chronic degenerative diseases such as atherosclerosis [2,4], cancer [5], Alzheimer’s, Parkinson’s [6] and rheumatoid arthritis [1].

The design of new MPO inhibitors has been based on their diverse modes of action such as complex II accumulation, suicide substrates and reversible union to the native enzyme. There are some MPO inhibitors, such as 5-aminosalicylic acid (5-ASA) [7], dapsone, tryptamines and indomethacin, which are considered as inhibitors that cause the accumulation of complex II (peroxidase deficient) [8,9]. Therefore, in order to promote complex II accumulation, these types of inhibitors first react with complex I; the latter being more reactive than the former with two and one oxidation equivalents, respectively, compared to the native MPO [10]. In contrast, suicide substrates such as 4-aminobenzoic acid hydrazide (4-ABAH), benzoic acid hydrazide (BAH) and 2-thioxanthins can be categorized as irreversible inhibitors, which either destroy or modify the covalent bond of the heme group present in the MPO [11]. On the other hand, reversible inhibitors such as salicylhydroxamic acid (SHA) and aromatic hydroxamates compete with the substrate for the active site of the enzyme but without modifying it.

Chronic degenerative diseases have long been associated with oxidative stress, mainly derived from the enzymatic production of reactive oxygen species (ROS) [12,13]. Generally, synthetic molecules designed to counteract the free radical effects are derived from compounds of natural origin and some are structurally similar. Polyphenols constitute a class of antioxidants which are found in the daily diet, commonly known as phytochemicals, where hydroxycinnamic acids are found. Caffeic, ferulic and isoferulic acids are synthesized within some plants such as coffee, cinnamon, citrus fruits and grapes, among others [14]; they are also found in wines [15]. Some of the reported pharmacological properties of cinnamic acid and its derivatives have anti-inflammatory, antimicrobial, antimalarial [16], antidepressant [17], anticancer and antioxidant activities, as well as neuroprotective effects [18]. 

**Figure 1:**
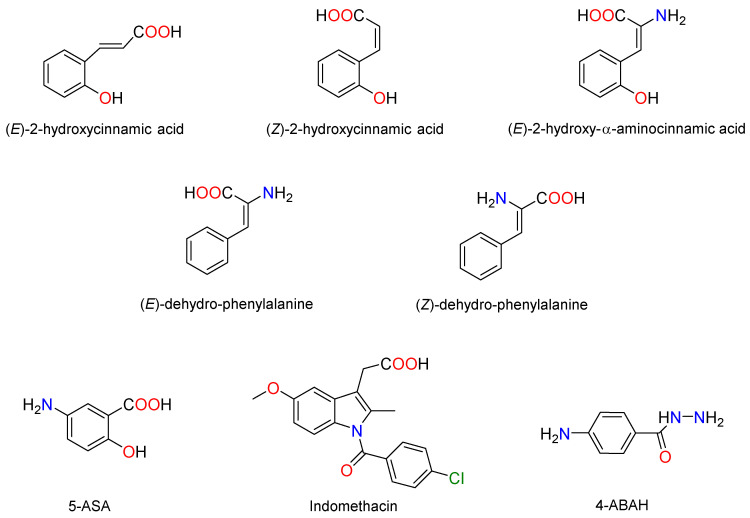
Structures of (*E*)- and (*Z*)-2-hydroxycinnamic acids, (*E*)-2-hydroxy-α-aminocinnamic acid, (*E*)- and (*Z*)-dehydro-phenylalanine, 5-aminosalicylic acid (5-ASA), indomethacin and 4-aminobenzoic acid hydrazide (4-ABAH) studied herein.

The antioxidant properties of both cinnamic acid and its hydroxy derivatives can be explained in terms of two important structural features: the presence of a phenol moiety and the propenoic acid on the side chain. Both mentioned moieties have been shown to confer an improved electron delocalization of the backbone upon reaction with free radicals, thus avoiding the stage of radical propagation during the oxidation process [19]. The carboxylic acid functional group and the phenolic ring, in hydroxycinnamic acids, can be found in opposite positions or in the same plane around the double bond. In the first case, cinnamic acids are referred to as *E* and in the second case as *Z* isomers, Figure 1. 2-Hydroxycinnamic acids are a group of compounds of natural origin, very abundant in some foods and appreciated for their antioxidant properties which can be mainly found in the *E* configuration, whereas the *Z* configuration is scarce. As a natural product, (*Z*)-2-hydroxycinnamic acid has been isolated in very small quantities [20,21]. Recent studies in lung cancer showed that (*Z*)-cinnamic acid prevents metastasis to a greater extent than the (*E*)-cinnamic acid [22]. It is worth mentioning that the (*E*) isomers are more abundant in nature, presumably because they are more stable than the (*Z*) isomers [23]. There are reported methods for the synthesis of (Z)-2-hydroxycinnamic acids, but the yields are low, at 5–10%, and purification by chromatography is necessary, with the main product being coumarin [24].

Nowadays, the pharmaceutical industry has integrated green chemistry into its processes to obtain products free of solvents and heavy metals [25]. One of the modern green methodologies is microwave-assisted chemical synthesis, which uses the property of polar molecules to transform electromagnetic energy into heat to save time and energy, generating fewer by-products and therefore improving yields, compared to conventional methods [26].

Herein, we present the synthesis of a family of (*E*)-2-hydroxy-α-aminocinnamic acid derivatives obtained through microwave-assisted chemical synthesis in good yields, Figure 1. These compounds are analogous to dehydro-amino acids which are highly valuable synthetic precursors [27] and particularly to (*E*)-dehydro-phenylalanine whose (*Z*) isomer has recently been used as hydrogelator [28]. In this work, the title compounds have been envisaged as antioxidants due to their structural analogy with (*Z*)-2-hydroxycinnamic acid. The structures of the abovementioned compounds and that of contrasting/inhibitory compounds used in the assays are depicted in Figure 1. Their antioxidant capabilities were evaluated using the 2,2-diphenyl-1-picrylhydrazyl (DPPH) and 2,2-azino-bis(3-ethylbenzothiazolin)-6-sulfonic acid (ABTS) methods, as long as molecular orbital calculations at the B3LYP/6–31 + G(d,p) level of theory allowed us to rationalize their mechanism of action. Selected compounds exhibited in vitro inhibitory activity against MPO whose interaction in the binding site was modeled by docking calculations. Finally, the cytotoxicity of this set of compounds was evaluated in a 3-(4,5-dimethylthiazol-2-yl)-2,5-diphenyltetrazolium bromide (MTT) assay.

## 2. Results and Discussion

### 2.1. Chemistry

Cinnamic acid derivatives **2a**–**j** were obtained in moderate to good yields (51–95%) from the corresponding 3-acetamidocoumarins **1a**–**j** [29]. Compounds **1a**–**j** were synthesized following a reported method [30] with modifications in the quantities of the reagents used and reaction time and starting from glycine instead of *N*-acetylglycine, resulting in higher yields (62–86%) than those reported elsewhere (40–69%) [31].

**Scheme 1:**
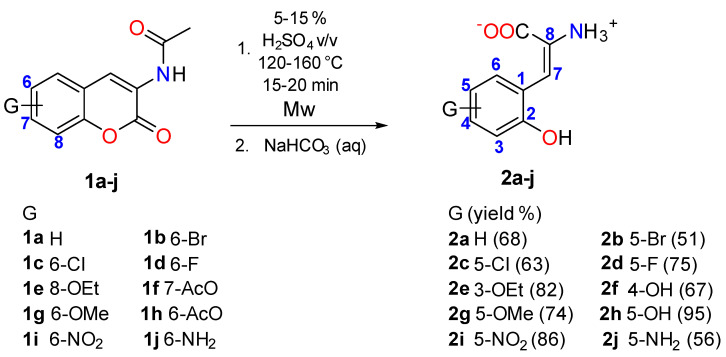
Microwave-assisted hydrolysis of acetamidocoumarins **1a**–**j** to produce (*E*)-2-hydroxy-α-aminocinnamic acids **2a**–**j**.

The general procedure to obtain **2a**–**j**, as switterions, is shown in Scheme 1, which consists of the microwave-assisted acid hydrolysis of the 3-acetamidocoumarins **1a**–**j**, followed by neutralization with NaHCO_3_. The reaction conditions were optimized for each compound since they depend on the nature of the substituent. The concentration of the H_2_SO_4_ aqueous solution, temperature and reaction time are the main factors that determine the progress of the reaction. 

**Figure 2:**
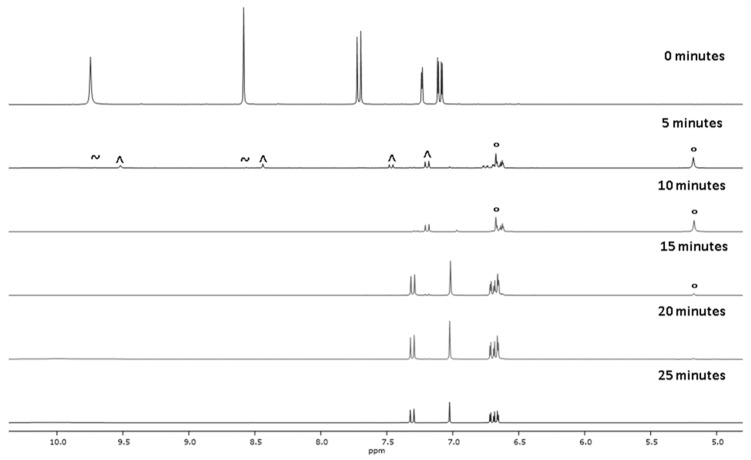
^1^H-NMR spectra (DMSO-d6) showing the effect of reaction time on the conversion of the starting compound **1f** (~) to **2f**, at a reaction temperature of 120 °C and 5% *v*/*v* of H_2_SO_4_ in water. The intermediates **I**–**f** (^) and **II**–**f** (°) are observed in 5 min of reaction time.

**Scheme 2:**
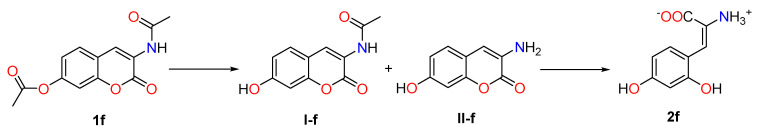
Observed intermediates **I**–**f** and **II**–**f** in the acid hydrolysis of **1f** to **2f**.

The ^1^H-NMR spectra of the isolated reaction mixtures, for the conversion of compound **1f** to **2f** at 120 °C and 5% *v*/*v* concentration of H_2_SO_4_ in water, with increasing reaction times, are shown in Figure 2. The complete conversion required 15 min of reaction at the specified conditions, however, this set of spectra allows us to observe the reaction intermediates. After 5 min of reaction time, the hydrolyzed ester **I**–**f** and the aminocoumarin **II**–**f** are observed. The mixture progresses to the complete lactone ring opening, leading to compound **2f**. The above result confirms that compound **2f** can also be obtained starting from the corresponding aminocoumarin, as sketched in Scheme 2. A summary of the optimized reaction conditions is listed in Table 1. In general, milder conditions are required for acetamidocoumarins substituted with electron-donating groups in comparison to those substituted with electron-withdrawing groups.

One of the characteristic structural features of compounds **2a**–**j** is the ^1^H-NMR signal of the vinyl proton H7, as shown in Scheme 1. It appears in the 6.95–7.26 ppm range as a singlet, which in the corresponding spectra of the starting **1a**–**j**, is shifted to higher frequencies, usually beyond 8.60 ppm [32] and in the aminocoumarins it appears at lower frequencies in the 6.59–6.71 ppm range [29]. The synthesis of 3-aminocoumarins, by the acid hydrolysis of 3-acetamidocoumarins, has been reported elsewhere [29], using H_2_SO_4_ solution (70%) by both microwaves and reflux. In this context, compounds **2a**–**j** are proposed as the natural final product of hydrolysis. However, we observed by ^1^H-NMR that compounds **2a**–**j** are cyclized with dehydration in highly concentrated H_2_SO_4_ solutions to regenerate the corresponding 3-aminocoumarin (Figures S1 and S2). This transformation requires the phenolic –OH and the –COOH groups on the same side around the double bond to perform the lactone ring cyclization. This experimental behavior supports the (*E*) configuration of compounds **2a**–**j**. The fact that the synthesis of **2a**–**j** was carried out successfully even though the (*Z*) derivatives of the cinnamic acid are reported to naturally occur in low yields or with little stability [21,22,24] points to the amino group as the structural factor that provides the observed stability of **2a**–**j**, particularly allowing the formation of the switterionic form which prevents lactonization to the corresponding aminocoumarin. 

### 2.2. DPPH and ABTS Free Radical-Scavenging Activity

**Figure 3:**
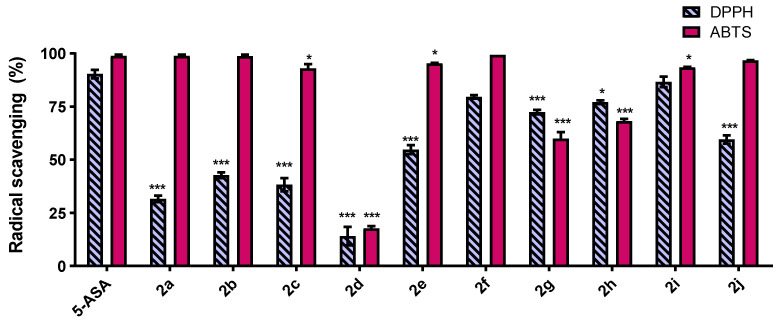
Comparison of the radical-scavenging activity of compounds **2a**–**j** vs. 5-ASA at a concentration of 0.32 mM and 0.04 mM in the DPPH and ABTS assays, respectively (* *p* < 0.05, *** *p* < 0.0001). The assays were performed in triplicate. DPPH RSA (%): 31.6 ± 2.6 (**2a**), 42.7 ± 2.4 **(2b**), 38.3 ± 5.4 (**2c**), 14.1 ± 7.5 (**2d**), 54.8 ± 3.7 (**2e**), 79.6 ± 1.4 (**2f**), 72.5 ± 1.8 (**2g**), 77.1 ± 1.5 (**2h**), 86.7 ± 4.3 (**2i**), 59.5 ± 3.4 (**2j**) and 90.4 ± 3.4 (5-ASA). ABTS RSA (%): 98.9 ± 1.3 (**2a**)**,** 98.7 ± 1.2 (**2b**), 93.1 ± 3.4 (**2c**), 17.7 ± 2.0 (**2d**), 95.4 ± 0.4 (**2e**), 99.4 ± 0.0, (**2f**), 60.0 ± 5.3 (**2g**), 68.3 ± 1.8 (**2h**), 93.5 ± 0.4 (**2i**), 96.8 ± 0.3 (**2j**) and 98.9 ± 1.3 (5-ASA).

The antioxidant activity of the cinnamic derivatives **2a**–**j** was evaluated using DPPH and ABTS tests, using 5-ASA as the reference compound. These tests are widely used to evaluate the removal of free radicals from natural and synthetic compounds; the results of both tests are depicted in Figure 3. In general, the antioxidant capacity evaluated through the DPPH test, using the highest tested concentration of 0.32 mM (see the complete results in Figures S3 and S4), decreased in the following order: 5-ASA = **2i** > **2f** = **2h** > **2g** > **2j** > **2e** > **2b** = **2c** > **2a** > **2d**. The radical scavenging activity (RSA) of compound **2i** is the same of 5-ASA (*p* < 0.0001) and compounds **2f** and **2h** reach up to 77% RSA, becoming almost as good as 5-ASA. The value of DPPH RSA of 5-ASA is similar to the reported values of 85–90% at 0.1–1.0 mM concentrations [33] and 80–85% at 0.20–0.41 mM concentrations [9]. The performances of compounds **2f**, **2h** and **2i** in the DPPH test are comparable to resveratrol–salicylate analogs (90% RSA, 0.20 mM) [34] and caffeic acid (94% RSA, 0.11 mM) [35], the latter being considered by some as the best cinnamic acid antioxidant [36]. 

On the other hand, compounds **2a**, **2b**, **2f** and **2j** exhibited ≈ 100% of RSA at 0.04 mM in the ABTS test, being as active as 5-ASA (*p* < 0.0001). The herein obtained value of ABTS RSA of 5-ASA is similar to the reported value of 98% at 0.05 Mm [9]. Compounds **2c**, **2e** and **2i** are within the 93.1–95.4% range, **2g** and **2h** are in the 60.0–68.3% range and the less effective **2d** has 17.7% RSA (see the complete results in Figures S5 and S6). Therefore, their performance in the ABTS test is better than the reported values for caffeic (93% RSA, 0.14 mM) [35] (45% RSA, 0.01 mM), sinapic (50%, 0.01 mM) and ferulic (40%, 0.01 mM) acids [37]. 

**Figure 4:**
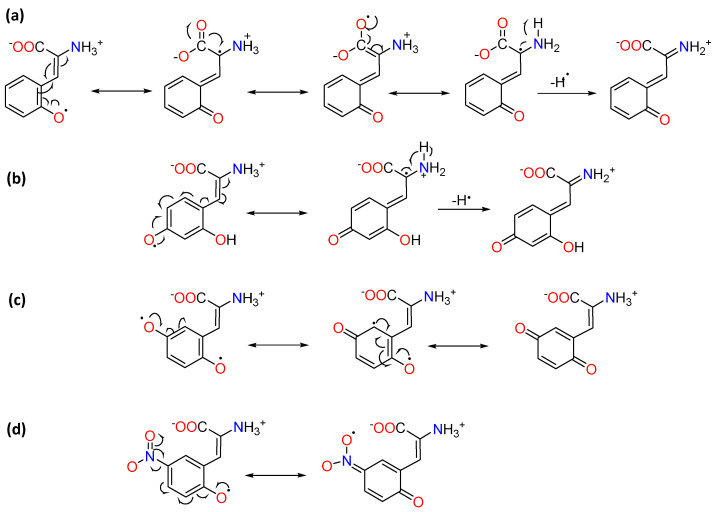
Resonant contributors to the stabilization of free radicals in compounds (**a**) **2a**, (**b**) **2f**, (**c**) **2h** and (**d**) **2i**.

Compounds **2f**–**j** show performances higher than 60% in both the DPPH and ABTS antioxidant tests; all of them are substituted with electron-donating groups with the exception of compound **2i** (5-NO_2_). In general, the RSAs of compounds **2a**–**j** are related to the possibility of π-type delocalization of the unpaired electron in the whole structure. In particular, the role played by the exocyclic conjugated double bond, carboxylic acid and the –NH_2_ group in the stabilization of the 2-OH radical is depicted in Figure 4a. Further, the highest antioxidant ability of **2f** is explained by the radical delocalization of the 4-OH group into the α-aminopropenoic fragment, Figure 4b. Compound **2h** is structurally comparable with synthetic salicylate derivatives whose RSA performance was demonstrated to depend on the 1,4-disposition between the two –OH groups in the molecule [33,34]. The stabilizing resonance structures, including the oxidation products of compound **2h**, are depicted in Figure 4c, ending with the formation of quinoid type products which can act as cytotoxic agents in diseases such as cancer, as reported for caffeic acid [38]. The results herein obtained are in agreement with those which point out that at last two phenolic –OH groups are required to confer antioxidant properties, but this quality is more dependent on their relative disposition [36]. Finally, the RSA of compound **2i**, substituted with the strong electro-withdrawing nitro group, is due to the increased acidity of the 2-OH group, facilitating the radical formation and also stabilizing it, Figure 4d. It is worth noting that the fluorine atom (**2d**) has the worst RSA in both DPPH and ABTS tests, in contrast to theoretical predictions (*vide infra*). This behavior could be associated with the high electronegativity of the fluorine atom, accompanied by its limited electronic delocalization possibilities.

### 2.3. Molecular Orbital Calculations and Antioxidant Mechanism 

The DPPH and ABTS tests are useful colorimetric probes accepting hydrogen atoms or electrons supplied by the antioxidant compounds. However, none of them allow discriminating between the hydrogen atom transfer (HAT) and single electron transfer (SET) as the most accepted mechanisms for phenols [39]. In this context, theoretical calculations at the B3LYP/6–31 + G(d,p) level of theory were performed in order to gain a better understanding of the operating antioxidant mechanism of compounds **2a**–**j**. The bond dissociation enthalpy of the 2-OH group (BDE_OH_), the ionization potential (IP), E_H_ (energy of the HOMO), E_L_ (energy of LUMO) and ΔE_H-L_ (HOMO-LUMO gap) in vacuum were calculated. The results are shown in Table 2. 5-ASA was used as the reference compound to determine the reliability of our calculations. All the calculated values for 5-ASA are in agreement with the reported ones [33]. 

BDE_OH_ is related to the HAT mechanism. It describes the thermodynamic stability of the O–H bond and small BDE_OH_ values are associated with increased susceptibility to homolytic O-H bond dissociation. The calculated BDE_OH_ values for compounds **2a**–**2j** are in the 52.99–81.13 kcal mol^−1^ range. The smallest value of 52.99 kcal mol^−1^ associated with the bromine compound **2b** is underestimated and therefore omitted from discussion. Then, compounds **2d** > **2a** > **2e** > **2h** > **2f** can be ranked as the most active (BDE_OH_ values in the 61.95–65.50 range), followed by the less active **2g** > **2c** > **2i** > **2j** (BDE_OH_ values in the 73.01–81.13 range) through the HAT mechanism. The values for the most active group are smaller than those reported for caffeic acid (72.25–73.95 kcal mol^−1^), ferulic acid (81.36–75.08 kcal mol^−1^) and *p*-coumaric acid (81.18 -79.86 kcal mol^−1^) [40,41] and even smaller than ascorbic acid (75.4 kcal mol^−1^) [42]. As far as we are concerned, these values are among the smallest reported for antioxidant compounds [43], indicating a great propensity to react by the HAT mechanism. The OR (R = H, Me, Et) groups favor small BDE_OH_ values of the 2-OH: the 5-OH (**2h**) group is better than 4-OH (**2f**) and the OR group is also more favorable in C-3 (**2e**) than in C-5 (**2g**). These structure trends are in agreement with those observed by others in hydroxycinnamic acids [44]. 

IP describes the ability of compounds in electron donation, and smaller values of IP are related to a more active SET mechanism [45]. In this context, compounds **2a**–**2j** can be sorted into three groups according to their calculated IP values. The most active group through the SET mechanism is represented by **2f** > **2e** > **2h** > **2a**, a moderately active group by the halogen substituted compounds **2b**-**2d** and the less active group by **2j** > **2i** > **2g**, showing IP values in the 135.67–138.84, 141.24–145.54 and 150.22–156.95 kcal mol^−1^ ranges, respectively. The IP values of the first group of compounds are similar to *p*-coumaric acid (130.43 kcal mol^−1^) and other cinnamic acids [44], but smaller than ascorbic acid (152.0 kcal mol^−1^) or cinnamaldehyde (154.9 kcal mol^−1^) [46], pointing to the good propensity of **2a**, **2e**, **2f** and **2h** to act through the SET mechanism.

In addition, E_H_, E_L_ and ΔE_H-L_ (HOMO–LUMO gap) values are related to the chemical reactivity of the molecule. The lower the ΔE_H-L_ value, the easier the electronic transition from ground state to excited state and the higher the chemical reactivity. The calculated ΔE_H-L_ value of compound **2i** is the smallest (3.92 eV), but comparable to the value of 5-ASA (3.94 eV) and sinapic acid (3.8 Ev) [45], whereas the ΔE_H-L_ values of the rest of the compounds are in the 4.53–4.81 eV range, slightly higher than those calculated for natural hydroxycinnamic acids (4.1–4.5 eV). 

In general, the calculated BDE_OH_ and IP values of compounds **2a**–**j** are in agreement with a greater propensity to react by the HAT mechanism. However, compounds **2a**, **2e**, 2**f** and **2h** are predicted to be the most active by either of the two mechanisms with larger stabilities than natural hydroxycinnamic acids.

**Figure 5:**
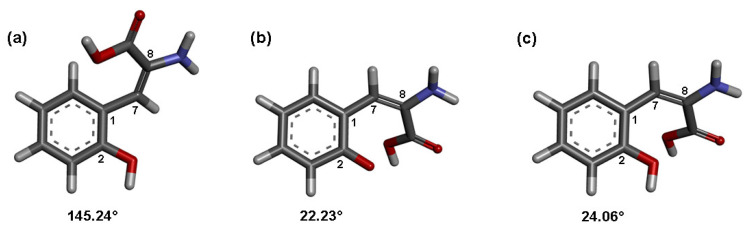
Predicted conformations of (**a**) **2a**, (**b**) **2a** radical and (**c**) **2a** cation radical. The torsion angle C2-C1-C7-C8 is shown.

Finally, it is worth mentioning that theoretical calculations predict a strong change in the conformation adopted by the α-aminopropenoic acid arm when radicals are formed. The C2-C1-C7-C8 torsion angle changes from the 143–147° range in **2a**–**j** to the 20–34° range in the radicals. Then, the planar α-aminopropenoic acid arm and the bent conformation adopted by radicals and cation radicals strongly resemble the structure of the 3-aminocoumarins, favoring the delocalization of the nitrogen atom lone pair to the phenyl ring which might contribute to their stabilization, Figure 5 [45]. A complete list of C2-C1-C7-C8 torsion angles is given in Table S1.

### 2.4. Docking of ***2a**–**j*** with MPO

Molecular docking was performed with the aim to support the choice of MPO. Three different conformers of the MPO from molecular dynamics (MD) simulation were used [47]. The validation of the method was carried out with *N*-acetylglucosamine, reaching an RMSD value of 1.194 Å in agreement with accepted values [48] (Figure S7). The calculated binding free energy (ΔG) and interactions in the binding site are listed in Table 3. In general, the ΔG values are more favorable at the MPO conformation adopted at 10 ns than at 5 or 0 ns (native structure). The calculated ΔG values (kcal mol^−1^) are in the −6.5 to −6.0 range. The largest values correspond to those compounds bearing halogens **2b** (5-Br) and **2c** (5-Cl). This result contrasts with reported results which mention that halide substituents in tested molecules to inhibit MPO have no effect on it [49]. Docking calculations predict that compounds **2a**–**j** bind to the MPO, forming complexes as stable as those of 4-ABAH, a well-known irreversible inhibitor, and 5-ASA, the reference compound. 

**Figure 6:**
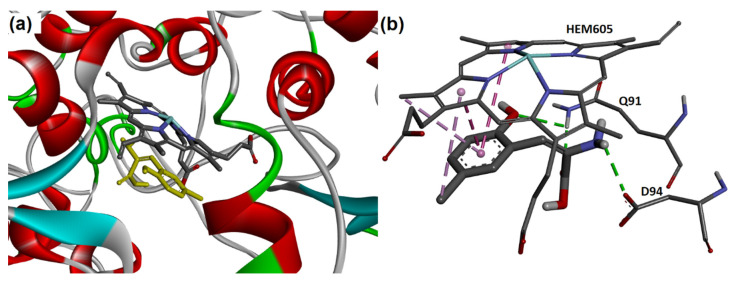
(**a**) 3D structure of one homodimer of the MPO forming a complex with compound **2c**. (**b**) The main hydrogen bonding interactions, between the carboxylic acid and amino groups with the aa residues glutamine (Q91) and aspartic acid (D94), respectively, in addition to the π-stacking interaction between 2-hydroxyphenyl ring with the HEM605, are shown.

In previous studies [47], the enzyme-binding sites were identified as being formed by the amino acid (aa) residues Q91, H95, R239, D94, F99, E242, R333, F366, F407 and HEM605, where Q91, H95, R239 and four water molecules make up the distal cavity of the MPO, associated with the halogenation cycle. Docking calculations predict that compounds **2a**–**j**, 4-ABAH and 5-ASA interact with the prosthetic heme group (HEM605) and also with the aa residues present in both the main binding site and the distal cavity: Q91, R239, D94, E242 and M243. These results are in agreement with those obtained in silico with triazolopyrimidines which bind into the active site in a reversible manner, avoiding the oxidation of the halides by the MPO [50]. The 3D structure of one homodimer of the MPO forming a complex with compound **2c** is depicted in Figure 6, where the type of non-covalent interactions is appreciated. 

### 2.5. MPO Enzymatic Activity of Peroxidation and Chlorination

**Figure 7:**
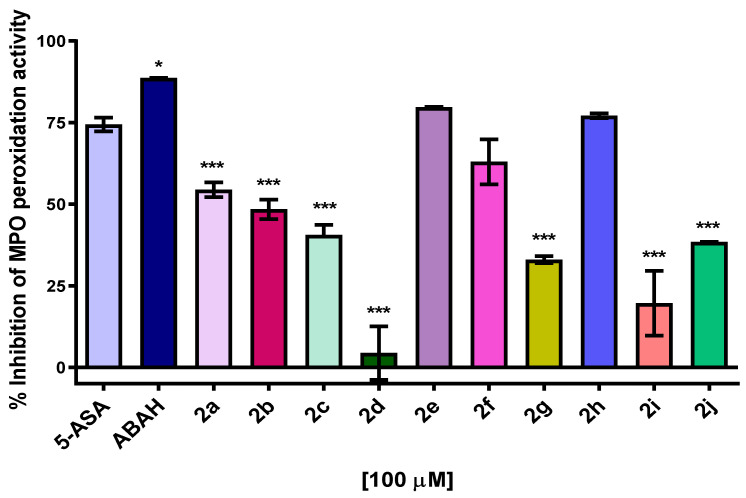
Comparison of the MPO peroxidation inhibitory activity of compounds **2a**–**j** vs. 5-ASA and 4-ABAH at 100 µM concentration (* *p* < 0.05, *** *p* < 0.001). The assays were performed in triplicate. Peroxidation inhibition values (%): 54.4 ± 3.1 (**2a**), 48.4 ± 4.1 (**2b**), 40.5 ± 4.4 (**2c**), 4.3 ± 0.1 (**2d**), 79.7 ± 6.6 (**2e**), 63.0 ± 1.5 (**2f**), 33.0 ± 1.1 (**2g**), 77.1 ± 3.9 (**2h**), 19.6 ± 0.1 (**2i**), 38.3 ±0.1 (**2j**) and 74.4 ± 2.9 (5 − ASA).

Peroxidation and chlorination enzymatic activities of the MPO, in the presence of compounds **2a**–**j**, were tested in vitro. The results are shown in Figure 7. All compounds, with the exception of **2d**, inhibit the peroxidation activity of the MPO. An inhibition larger than 50% is exhibited by compounds **2a**, **2e**, **2f** and **2h** at 100 μM. Particularly, the inhibition of MPO by **2e** and **2h** reaches values as high as 79%, comparable to 5-ASA. The IC_50_ of this set of compounds was estimated using a semi-logarithmic correlation of % inhibition against the log of the concentration (3, 6, 12, 25, 50, 100, 200, 400 μM), the dose–response graphs are depicted in Figure S8. There are no significant differences between the IC_50_ values (µM) of **2f** (30 ± 5), **2h** (26 ± 2) and indomethacin (29 ± 2), whose IC_50_ values are smaller than those corresponding to **2a** (45 ± 4), **2e** (43 ± 4) and 5-ASA (39 ± 2). These results are in agreement with those obtained in DPPH and ABTS tests.

**Figure 8:**
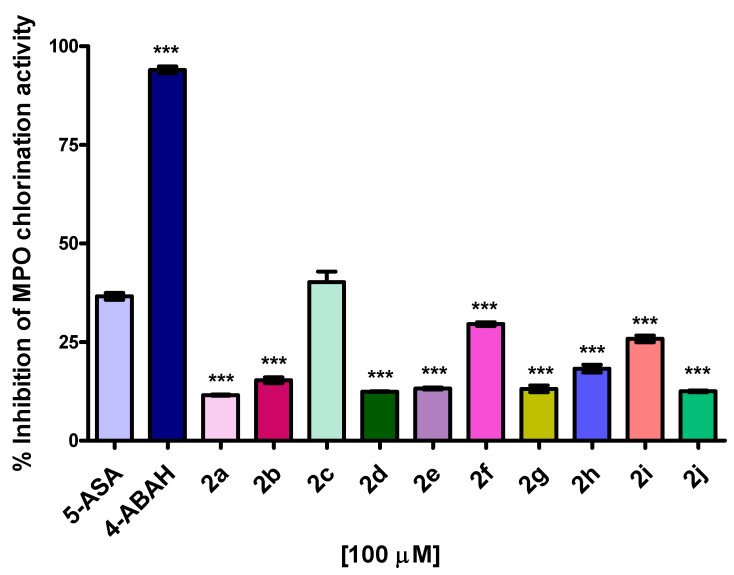
Comparison of the MPO chlorination inhibitory activity of compounds **2a**–**j** vs. 5-ASA and 4-ABAH at 100 µM concentration (*** *p* < 0.001). The assays were performed in triplicate. Chlorination inhibition values (%): 11.6 ± 0.1 (**2a**), 15.3 ± 0.4 (**2b**), 40.2 ± 1.2 (**2c**), 12.4± 0.1 (**2d**), 13.2± 0.1 (**2e**), 29.6 ± 0.3 (**2f**), 13.1 ± 0.5 (**2g**), 18.3 ± 0.6 (**2h**), 25.8 ± 0.5 (**2i**), 12.5 ± 0.1 (**2j**) and 36.6 ± 0.5 (5 − ASA).

The chlorination test shows that compounds **2c**, **2f** and **2i** exhibit a significant decrease in the enzymatic activity of chlorination, in the range of 20–40%, with **2c** being the best MPO-inhibiting compound, showing the same activity as 5-ASA at 100 μM, Figure 8. These results indicate that the analyzed compounds, including 5-ASA, are not specific for MPO inhibition, therefore, they could be good candidates for other peroxidases which do not carry out the halogenation cycle, such as COX inhibitors.

### 2.6. Cell Viability 

**Figure 9:**
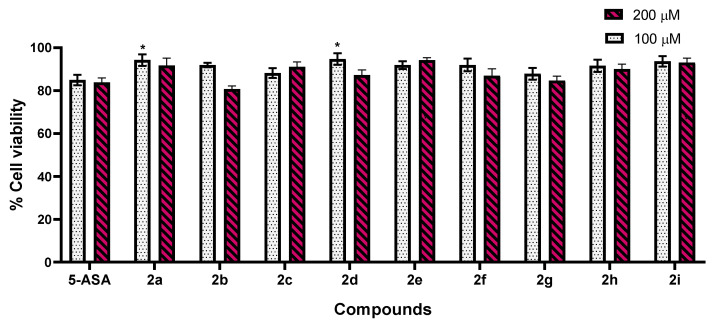
Percentage of cell viability of NIH/3T3 cells after treatment with compounds **2a**–**i** at 100–200 µM. * *p* < 0.05.

Compounds **2a**–**j** exhibit antioxidant capabilities and MPO inhibitory activity. Thus, in order to gain insight into the cytotoxic potential of these derivatives, they were tested at 12.5–200 μM on the fibroblast cell line NIH/3T3. The cell viability was measured by performing an MTT assay with the maximum pattern cell activity occurring at 100% of MTT reduction. Results at 100 and 200 µM are shown in Figure 9. The complete data can be found in Table S2. In general, the cytotoxicity of compounds **2a**–**2i** is low. The bromine derivative **2b** was found to be the most cytotoxic, diminishing the cell viability up to 19% at 200 µM. The rest of the compounds maintain the cell viability above 85%, and the effect is unrelated to the concentration. 

## 3. Materials and Methods 

Substituted salicylaldehydes, sodium acetate, glycine, acetic anhydride, 2,2-diphenyl-1-picrylhydrazyl (DPPH), 2,2-azino-bis(3-ethylbenzothiazolin)-6-sulfonic acid (ABTS), α-phenyl-*N*-*tert*-butylnitrone (PBN), 5-aminosalicylic acid (5-ASA), hydrogen peroxide solution 30 wt % in H_2_O (ACS reagent), taurine (≥99% purity), DTNB (≥99% purity), *ortho*-dianisidine and solvents were analytical grade and used as received (Sigma-Aldrich). Myeloperoxidase of human polymorphonuclear leukocytes (EC number 1.11.1.7, >95% purity) from Millipore^®^ Merk was used. The synthetic procedures and characterization of compounds **1a**–**j** are described in the Supplementary Materials.

The microwave-assisted synthesis of compounds **2a**–**j** was carried out in a Monowave 300 synthesis reactor from Anton Paar operating at 850 W. IR spectra were recorded neat at 25 °C with a Perkin Elmer Spectrum GX series with an FT system spectrophotometer using the ATR device. Melting points were measured in an Electrothermal IA 91000 device. ^1^H and ^13^C NMR spectra were acquired on a Varian Mercury NMR spectrometer operating at 300 MHz (^1^H, 300.08; ^13^C, 75.46 MHz) or Bruker Avance DPX-400, using DMSO-d6 as a solvent. All chemical shift values (δ) are reported in parts per million (ppm), using as a reference the residual solvent peak (^1^H, δ 2.50; ^13^C, δ 39.52) and coupling constants *^n^J*(H–H) in Hz. Multiplicity of the signals are expressed as: s (singlet), d (doublet), t (triplet), q (quartet), m (multiplet) or br (broad) (Figures S9–S38).

### 3.1. Synthetic Procedures and Characterization of Compounds ***2a**–**j***

*(E)-(2-hydroxy)-α-aminocinnamic acid* (**2a**). First, 0.300 g of *N*-(2-oxo-2*H*-chromen-3-yl) acetamide (1.48 mmol), **1a**, were dissolved in 18 mL of a recently prepared aqueous H_2_SO_4_ solution of 15% (*v*/*v*) inside a 30 mL microwave vial. The reaction was allowed to proceed for 15 min at 120 °C and 1200 rpm. Thereafter, the reaction was neutralized with NaHCO_3_ (0.70 g), and the resulting solid was then filtered and washed with distilled water (10 mL) to afford **2a** as a white powder in 68% yield (0.180 g, 1.00 mmol), m.p. 202 °C, Rf: 0.68 hexane/ethyl acetate (AcOEt) 1:1. ^1^H-NMR δ: 7.46 (dd, 1H, ^3^*J* = 7.5, H6), 7.26 (m, 2H, H5,3), 7.23 (m, 1H, H4), 6.95 (s, 1H, H7). ^13^ C-NMR δ: 161.0 (C=O), 148.7 (C2), 146.5 (C8), 126.0 (C4), 125.7 (C6), 124.9 (C5), 123.12 (C1), 115.9 (C3), 112.3 (C7). IR (cm^−1^): 3043 (N-H), 1681 (C=O), 1456, 735.

*(E)-(5-Bromo-2-hydroxy)-α-aminocinnamic acid* (**2b**). The synthesis was carried out as previously indicated for derivative **2a**, starting from 0.300 g (1.06 mmol) of **1b** under the following conditions: 160 °C for 20 min, to yield 0.140 g (0.540 mmol, 51%) of a brown solid, m.p. 243 °C, Rf: 0.76 hexane/AcOEt 1:1. ^1^H-NMR δ: 7.82 (d, 1H, ^4^*J* = 2.3, H6), 7.52 (dd, 1H, ^3^*J* = 8.6, ^4^*J* = 2.5, H4), 7.31 (d, 1H, ^3^*J* = 8.7, H3), 7.09 (s, 1H, H7) 10.7 (s, 1H, OH). NMR ^13^C δ: 158.3 (C=O), 148.6 (C2), 143.0 (C8), 130.4 (C4), 128.8 (C6), 123.3 (C1), 118.3 (C5), 116.8 (C3), 114.0 (C7). IR (cm^−1^): 3355 (N-H), 1690 (C=O), 1384, 771. 

*(E)-(5-Chloro-2-hydroxy)-α-aminocinnamic acid* (**2c**). The synthesis was carried out as previously indicated for derivative **2b**, starting from 0.300 g (1.26 mmol) of **1c** to yield 0.170 g (0.790 mmol, 63%) of a brown solid, m.p. 252 °C, Rf: 0.81 hexane/AcOEt 1:1. ^1^H-NMR: δ 10.7 (br, 1H, OH), 7.64 (d, 1H, ^4^*J* = 2.0, H6), 7.09 (s, 1H, H7), 7.39 (m, 2H, H4, 3). ^13^ C-NMR: δ 158.3 (C=O), 148.1 (C2), 143.0 (C8), 128.9 (C5), 127.6 (C4), 125.8 (C6), 122.8 (C1), 117.9 (C3), 114.1 (C7). IR cm^−1^: 3381 (N-H), 1720 (C=O), 1518, 919, 835.

*(E)-(5-Fluoro-2-hydroxy)-α-aminocinnamic acid* (**2d**). It was synthesized as indicated for compound **2b**, starting from 0.300 g (1.36 mmol) **1d** to yield 0.200 g (1.01 mmol, 75%) of a brown solid m.p. 242 °C, Rf: 0.84 hexane/AcOEt 1:1. ^1^H-NMR: δ 7.43 (dd, ^3^*J*_H-F_ = 9.1, ^4^*J* = 3, H6), 7.39 (dd, ^4^*J*_H-F_ = 5, ^3^*J* = 9.1, H3), 7.22 (ddd, ^3^*J*_H-F_ = 9.0, ^3^*J* = 8.6, ^4^*J* = 3, H4), 7.08 (s, 1H, H7). ^13^ C-NMR: δ 160.1 (C=O), 158.5 (C2), 145.8 (C8), 143.2 (C5), 122.6 (^3^*J*_C-F_ = 10.3, C1), 117.8 (^3^*J*_C-F_ = 9.1, C3), 114.8 (^2^*J*_C-F_ = 24.8, C4), 114.3 (C7), 112.0 (^2^*J*_C-F_ = 25, C6). IR cm^−1^: 3115 (N-H), 1676 (C=O), 1518, 748.

*(E)-(3-Ethoxy-2-hydroxy)-α-aminocinnamic acid* (**2e**). It was synthesized as indicated for compound **2a**, starting from 0.300 g (1.20 mmol) of **1e** to yield 0.220 g (0.990 mmol, 82%) of a brown solid, m.p. 139 °C, Rf: 0.70 hexane/AcOEt 1:1. ^1^H-NMR: δ 10.4 (br, 1H, OH), 7.16 (t, 1H, ^3^*J* = 8.1, H5)_,_ 7.07 (s, 1H, H7), 7.04 (m, 2H, H4, 6), 4.11(c, 2H, CH_2_, ^3^*J* = 7.0), 1.37 (t, 3H, CH_3_, ^3^*J* = 7.0).^13^C- NMR: δ 158.6 (C=O), 145.9 (C2), 142.3 (C8), 138.7 (C3), 125.0 (C6), 121.7 (C1), 118.1 (C5), 115.5 (C7), 111.4 (C4), 64.5 (CH_2_), 15.0 (CH_3_). IR cm^−1^: 3352 (N-H, 1692 (C=O), 1472, 764.

*(E)-(2,4-dihydroxy)-α-aminocinnamic acid* (**2f**). It was synthesized as indicated for compound **2a**, starting from 0.300 g (1.15 mmol) of compound **1f** and 18 mL of a 5% *v*/*v* H_2_SO_4_ solution, at 120 °C, 10 min to yield 0.180 g (0.920 mmol, 67%) of a brown solid, m.p. 272 °C, Rf: 0.77 hexane/AcOEt 1:1. ^1^H-NMR: δ 10.0 (br, 2H, OH), 7.35 (d, 1H, ^3^*J* = 8.5, H6)_,_ 7.06 (s, 1H, H7), 6.74 (dd, 1H, ^3^*J* = 8.5, ^4^*J* = 2.3, H5), 6.69 (d, 1H, ^4^*J* = 2.3, H3). ^13^C NMR: δ 159.2 (C=O), 158.2 (C2), 151.0 (C4), 139.1 (C8), 127.7 (C6), 116.6 (C7), 113.6 (C5), 112.7 (C1), 102.4 (C3). IR cm^−1^: 3278 (OH, N-H), 1696 (C=O), 1454, 826.

*(E)-(2-hydroxy-5-methoxy)-α-aminocinnamic acid* (**2g**). It was synthesized as indicated for compound **2a**, starting with 0.300 g (1.29 mmol) of **1g** to yield 0.200 g (0.95 mmol, 74%) of a brown solid. m.p. 154 °C, Rf: 0.67 hexane/AcOEt 1:1. ^1^H- NMR: δ 10.4 (br, 1H, OH), 7.24 (d, 1H, ^3^*J* = 9.0, H4)_,_ 7.05 (d, 1H, ^4^*J* = 3.2, H6), 6.98 (s, 1H, H7) 6.93 (dd, 1H, ^3^*J* = 8.8, ^4^*J* = 3.0, H3), 3.75 (s, 3H, CH_3_).^13^C- NMR: δ 159.4 (CO_2_), 156.3 (C2, 5), 143.6 (C8), 122.4 (C6), 116.9 (C7), 114.4 (C4), 114.2 (C1), 109.1 (C3), 56.0 (OCH_3_). IR cm^−1^: 3360 (N-H), 1680 (C=O), 1400, 765.

*(E)-(2,5-dihydroxy)-α-aminocinnamic acid* (**2h**). It was synthesized as indicated for compound **2a**, starting from 0.300 g (1.15 mmol) of **1h** to yield 0.200 g (1.02 mmol, 95%) of a brown solid, m.p. 211 °C. Rf: 0.79 hexane/AcOEt 1:1. ^1^H- NMR: δ 10.2, 9.5 (br, 1H each, OH), 7.14 (d, 1H, ^3^*J* = 8.7, H3)_,_ 7.00 (s, 1H, H7), 6.81 (d, 1H, ^4^*J* = 2.6, H6), 6.76 (dd, 1H, ^3^*J* = 8.8, ^4^*J* = 2.6, H4). ^13^C-NMR: δ 159.4 (C=O), 154.8 (C2), 143.3 (C8), 143.5 (C5), 122.0 (C1), 117.3 (C3), 116.3 (C4), 115.8 (C7), 111.5 (C6). IR cm^−1^: 3249 (OH, NH), 1690 (C=O), 1456, 766.

*(E)-(2-hydroxy-5-nitro)-α-aminocinnamic acid* (**2i**). It was synthesized as indicated for compound **2b**, starting from 0.300 g (1.21 mmol) of **1i** to yield 0.230 g (1.04 mmol, 86%) of a brown solid, m.p. 240 °C, Rf: 0.7 hexane/AcOEt 1:1. ^1^H-NMR δ 8.54 (d, 1H, ^4^*J* = 2.7, H6), 8.16 (dd, 1H, ^3^*J* = 8.9, ^4^*J* = 2.7, H4)_,_ 7.54 (d, 1H, ^3^*J* = 9.1, H3), 7.26 (s, 1H, H7). ^13^C NMR: δ 158.1 (CO_2_), 153.4 (C2), 144.5 (C8), 143.9 (C5), 122.9 (C4), 122.6 (C6), 122.3 (C1), 117.7 (C3), 114.3 (C7). IR cm^−1^: 3456 (N-H), 1717 (C = O), 1518, 1343, 772. 

*(E)-(5-amino-2-hydroxy)-α-aminocinnamic acid* (**2j**). It was synthesized as indicated for compound **2a**, starting from 0.300 g (1.37 mmol) of **1j** to yield 0.150 mg (0.770 mmol, 56%) of a brown solid, m.p. 246 °C, Rf: 0.43 hexane/AcOEt 1:1. ^1^H-NMR: δ 7.07 (d, 1H, ^3^*J* = 8.2, H3), 6.96 (s, 1H, H7), 6.67 (dd, 1H, ^3^*J* = 8.2, ^4^*J* = 2.3, H4), 6.61 (d, 1H, ^4^*J* = 2.3, H6). ^13^C NMR: δ 158.9 (CO_2_), 145.7 (C2), 141.5 (C8), 141.2 (C5), 121.0 (C7), 116.2 (C3), 115.4 (C1), 115.0 (C4), 108.9 (C6). IR cm^−1^: 3369 (N-H), 3225 (O-H), 1719 (C=O), 1654, 1409, 1200, 117, 1047, 873.

### 3.2. DPPH Assay (2,2-Diphenyl-1-picrylhydrazyl) 

One hundred microliters of DPPH 0.20 mM in absolute methanol and 100 μL of the appropriate compound (0.32, 0.16, 0.08, 0.04, 0.02, 0.01 mM final concentrations) dissolved in DMSO were poured into a 96-well plate. The mixtures were incubated for 30 min at room temperature and protected from light. The absorbance was recorded at 517 nm in a transparent 96-well test microplate (Multiskan-EX Thermo Scientific, Thermo Fisher Scientific, Waltham, MA, USA). The results are expressed as percentage of DPPH radical present for each concentration of derivatives. To determine the antioxidant activity of each compound, the percentage of the DPPH radical-scavenging activity was calculated by the following equation: [1 − (A_1_ − A_2_)/(A_DPPH_ − A_S_)] × 100, where: A_1_ = Absorbance of the compound with DPPH, A_2_ = Absorbance of the compound, A_DPPH_ = Absorbance of DPPH (diluted 1:1 with solvent) and A_S_ = Absorbance of DMSO.

### 3.3. ABTS Test (2,2-Azino-bis(3-ethylbenzothiazolin)-6-sulfonic Acid)

ABTS dissolved in water to a 7.00 mM final concentration was allowed to react for 16 h at room temperature in the dark with an aqueous solution of potassium persulfate 2.45 mM to produce the ABTS radical cation (ABTS^+ •^) before use. In a 96-well plate, 100 μL of the appropriate compound **2a**–**2j** (0.040, 0.020, 0.010, 0.005, 0.0025 and 0.0012 mM final concentrations) dissolved in water was mixed with either 100 μL of diluted ABTS^+ •^ solution or solvent. The reaction was allowed to proceed for 30 min at room temperature and was protected from light. The absorbance was recorded at 734 nm in a transparent 96-well test microplate (Multiskan-EX Thermo Scientific) [51].

The antioxidant activity was calculated as the percentage of the ABTS cationic radical-scavenging activity with the test compound, by the following equation: [1 − (A_1_ − A_2_)/(A_ABTS_ − A_S_)] × 100, where: A_1_ = Absorbance of the compound with ABTS; A_2_ = Absorbance of the compound with solvent, A_ABTS_ = Absorbance of ABTS (diluted 1:1 with solvent), A_S_ = Absorbance of DMSO. The reference used for DPPH and ABTS tests was 5-ASA. 

### 3.4. MPO Enzymatic Activity of Peroxidation

The enzymatic activity of peroxidation was conducted as has been described previously [52] with modifications. Briefly, hydrogen peroxide (30%) was mixed with *ortho*-dianisidine in phosphate buffer (PBS) 50 mM, pH 6.0 to a final concentration of 0.0050% (1632 μM) and 167 μg/mL (684 μM), respectively. In a 96-well plate, 5 μL of a stock solution of MPO (0.0126 units/μL) were mixed with 5 μL of PBS and 10 μL of the appropriate concentrated DMSO solution of compounds **2a**–**j**, 5-ASA or 4-ABAH (200, 100, 50, 25, 12.5, 6 and 3 mM final concentrations per well) and 180 μL of the *ortho*-dianisidine–hydrogen peroxide solution (616 μM/1469 μM final concentrations per well). Control wells were treated with 10 μL of DMSO. The reaction was allowed to react for 30 min and the absorbance was measured at 460 nm. This assay was performed in triplicate.

### 3.5. MPO Enzymatic Activity of Chlorination

The enzymatic activity of chlorination was determined by the production of HOCl, using the taurine–chloramine assay, where HOCl production is detected indirectly by oxidizing 5-thio-2-nitrobenzoic acid (TNB), following a reported procedure [53]. TNB was prepared starting from DTNB, and 20 mg of DTNB (1.0 mM) and 40 mg of NaBH_4_ (20 mM) were mixed in 50 mL of PBS at pH = 7.4 and incubated for 30 minutes at room temperature. Then, a dilution was made by taking 430 μL of the TNB solution and 4570 μL of PBS, whose measured absorbance at 412 nm of 0.88 units is equivalent to 1.36 mM of TNB. The assay was performed in a 96-well plate, and into each well were placed: 0.126 units of MPO (10 μL), 53 μL of PBS 10 mM at pH = 7.4 added to 300 mM NaCl, 7 μL of 0.15 M taurine (10 mM final concentration). Ten microliters of 2.0 mM DMSO solutions of compounds **2a**–**j**, 5-ASA or 4-ABAH (100 μM final concentration) and 7 μL of 0.1% hydrogen peroxide (10.3 mM final concentration) were incubated for 10 min. Control wells were treated with 10 μL of DMSO instead. The reaction was stopped with 7 μL of catalase 4 U/μL, 10.5 μL of TNB and 95.5 μL of water. The absorbance was measured at 412 nm after 10 min. 

### 3.6. Cell Viability

The cell line NIH/3T3 was used, following reported procedures [54,55]. The cells were cultured in Dulbecco’s modified Eagle medium (DMEM) with phenol red, supplemented with 10% fetal bovine serum (FBS) and 1% penicillin/streptomycin. All cells were cultured at 37 °C in a humidified incubator containing 5% CO_2_. The cells were detached using 1 mL of trypsin 1% and 4 mL of PBS-EDTA. Afterwards, the cells were counted, and seeded in 96-well plates at 10 × 10^3^ cells/well. After 24 h, the cells were treated with 12, 25, 50, 100 and 200 μM of each compound in a final volume of 100 μL (0.2% of DMSO), specific wells were dedicated for control cells treated only with medium or medium with DMSO. After 48 h, the medium was removed and replaced by 20 μL of PBS containing MTT (0.5 mg/mL), and the cells were incubated for 3 h, at 37 °C under CO_2_. After that, the BS/MTT was discarded, and the formazan crystals produced were solubilized with DMSO (100 μL/well). The optical density was measured at 550 nm using a microplate reader (Multiskan-EX Thermo Scientific). Results are expressed as percentage of cell viability relative to control. The experiments were performed in triplicate.

### 3.7. Docking MPO

Validation of the method was performed with *N*-acetylglucosamine, reaching an RMSD value of 1.194 Å [48]. Snapshots (0, 5 and 10 ns) of different conformations of MPO (PDB: 1DNU) were obtained from MD simulations [47]. All ligands were 3D prepared in ChemSketch and optimized using Gaussian 98 software employing the AM1 method. All possible torsions and partial charges were identified and calculated using AutoDock Tools 1.5.6 software. All polar hydrogens were incorporated to the protein along with the Kollman charges for all atoms. All other parameters were maintained at their default values. The protein binding and scanning sites were prepared using a GRID-based method. A box of 126 Å^3^ was employed with a spacing of 0.375 Å centered on the iron atom of the heme group using the conformation at 10 nm. All simulations employed the hybrid Lamarckian genetic algorithm with an initial population of 100 randomly placed individuals and 1 × 100 evaluations. Results were analyzed with Discovery Studio Visualizer 19.1 software, to obtain both the binding energy (ΔG) and the interactions of compounds (**2a**–**2j**) with amino acid residues of MPO.

### 3.8. Molecular Orbital Calculations

Calculations were performed as reported [56,57,58], and a brief summary is described. All molecules and radicals were initially modeled under the semi-empirical method PM3. Geometry optimizations and frequency calculations were performed using DFT calculations at the B3LYP/6–31 + G(d,p) level of theory for parent molecules **2a**–**j**, radicals and cation radicals. Vibrational frequencies were scaled by a factor of 0.973 to obtain the scaled zero-point energy. The thermal correction of the enthalpy was computed by a single-point calculation with B3LYP/6–31 + G(d,p) level of theory. The total enthalpy at 298.15 K was calculated as the sum of the thermal correction of the enthalpy and the total electronic energy. The binding dissociation enthalpy values of the O-H bond (BDE_OH_) were calculated according to the formula BDE_OH_ = H_R_ + H_H_ − H_P_, where H_R_ is the enthalpy of the radical generated by H-abstraction, H_H_ is the enthalpy of the H-atom and H_P_ is the enthalpy of the corresponding parent compound **2a**–**j**. The enthalpy value of −0.49764 Hartree was used for the hydrogen atom in all calculations of the BDE. The IP values were obtained according to the equation IP = E_R+_ − E_P_, where E_R+_ is the energy of the cation radical, E_P_ is the energy of the corresponding parent compound **2a**–**j**. All calculations were performed with the Gaussian 09 molecular package [59] in a computer with an unlocked AMD FX processor. All the calculations refer to the gas phase. The Z-matrices, total energy and imaginary frequencies are in the Supplementary Materials. 

### 3.9. Statistical Analysis

One-way ANOVA was used for all the assays and a maximum value of *p* < 0.05 was considered statistically significant. DPPH, ABTS, MPO enzyme activity of peroxidation and cell viability assays were performed in triplicate whereas the MPO enzyme activity of chlorination assay was performed in duplicate and the results were standardized and expressed as the mean ± standard error (S.E.). A Tukey post hoc test was used.

## 4. Conclusions

The microwave-assisted synthesis of a family of (*E*)-2-hydroxy-α-aminocinnamic acids, analogous to (*Z*)-2-hydroxycinnamic acids, was successfully achieved in moderate to good yields in a diluted H_2_SO_4_ aqueous solution. The results obtained in the present study have shown that five (**2f**–**j**) of the ten studied compounds can effectively scavenge DPPH and ABTS free radicals under in vitro conditions, with activities similar to those reported for caffeic acid. The conjugated α-aminopropenoic acid skeleton and the substituents on the phenyl ring play an important role in their observed antioxidant properties, with those with a second ED group being the most active. The propensity of compounds **2a**–**j** to react through the HAT mechanism rather than the SET mechanism was predicted from BDE_OH_, IP and ΔE_H-L_ theoretical calculations, with compounds **2a**, **2e,** 2**f** and **2h** being the most active by either of the two mechanisms. Furthermore, we have demonstrated that compounds **2a**, **2e**, **2f** and **2h** showed in vitro peroxidation inhibitory activity of MPO with IC_50_ values comparable to indomethacin and 5-ASA but only the chlorinated derivative **2c** was capable of decreasing the in vitro chlorination activity of the MPO by 40% while the cytotoxicity of the whole set of compounds was below 15% at 100–200 µM. Docking calculations allowed for establishing that both amino and carboxylic acid groups of the α-aminopropenoic acid arm are required for anchoring to the MPO active site, forming stable complexes comparable to 4-ABAH-MPO and 5-ASA-MPO. To the best of our knowledge, (*E*)-2-hydroxy-α-aminocinnamic acids have been synthesized for the first time with a reliable method and their antioxidant properties demonstrated, introducing the possibility to contrast the pharmacological properties between configurational isomers. Finally, the pharmacological properties of these compounds, related to the generation of oxidant species, will be the subject of further research. 

## Figures and Tables

**Table 1:** Optimized reaction conditions to obtain compounds **2a**–**2j** using microwaves.

Compounds	Reaction Conditions		
	Aq. H_2_SO_4_ conc. (% *v*/*v*)	Temperature (°C)	Time (min)
**2f**	5	120	15
**2a, 2e, 2g, 2h, 2j**	15	120	15
**2b, 2c, 2d, 2i**	15	160	20

**Table 2:** BDE_OH_, IP, E_H_, E_L_ and ΔE_H-L_ calculated values at B3LYP/6–31 + G(d,p) in vacuum of 5-ASA and compounds **2a**–**j**.

Comp.	BDE_OH_ (kcal mol^−1^)	IP (kcal mol^−1^)	E_H_ (eV)	E_L_ (eV)	ΔE_H-L_ (eV)
5-ASA [33]	92.60	165.87	−5.66	−1.81	3.85
5-ASA	95.44	165.71	−5.79	−1.85	3.94
**2a**	62.02	138.84	−5.80	−1.05	4.75
**2b**	52.99	141.24	−5.93	−1.33	4.60
**2c**	75.68	143.20	−5.90	−1.27	4.63
**2d**	61.95	145.54	−5.90	−1.24	4.66
**2e**	62.79	134.50	−5.76	−0.95	4.81
**2f**	65.50 55.14 ^a^	135.67	−5.61	−0.96	4.65
**2g**	73.01	156.95	−5.55	−1.02	4.53
**2h**	62.85 53.95 ^b^	136.42	−5.58	−1.05	4.53
**2i**	79.29	152.65	−6.42	−2.51	3.91
**2j**	81.13	150.22	−5.39	−0.78	4.61

^a^ 4-OH; ^b^ 5-OH.

**Table 3:** Values of ΔG (kcal mol^−1^) of the compounds **2a**–**j** in the 10 ns conformation of the MPO enzyme and their interactions with the amino acid residues.

Comp.	ΔG (kcal mol^−1^)	Interaction Residue
**4-ABAH**	−6.0	HEM605, Q91, D94, R239, M243
**5-ASA**	−6.1	HEM605, Q91, D94, R239, E242, M243, L246, H336
**2a**	−6.3	HEM605, Q91, D94, R239, M243, L246
**2b**	−6.5	HEM605, Q91, D94, R239, M243, L246
**2c**	−6.4	HEM605, Q91, D94, R239, M243, L246
**2d**	−6.3	HEM605, Q91, D94, R239, S240, M243, L246
**2e**	−6.3	HEM605, Q91, D94, R239, E242, M243, L246, H336, F365
**2f**	−6.1	HEM605, Q91, D94, R239, M243, L246, H336
**2g**	−6.2	HEM605, Q91, D94, R239, M243, L246
**2h**	−6.1	HEM605, Q91, D94, R239, E242, M243, L246, H336, F365
**2i**	−6.3	HEM605, Q91, D94, R239, M243
**2j**	−6.0	HEM605, Q91, D94, R239, M243, L246

## Data Availability

The data presented in this study are available on request from the corresponding author.

## Supplementary Materials

The following are available on line at https://www.mdpi.com/article/10.3390/ph14060513/s1, Figures S1 and S2: ^1^H-NMR spectra showing the effect of the H_2_SO_4_ concentration on the conversion of **1i** to **2i** and **1a** to **2a**; Figures S3 and S4: Percentage of DPPH RSA with compounds **2a**–**j**; Figures S5 and S6: Percentage of ABTS RSA with compounds **2a**–**j**; Figure S7: Validation of the molecular docking protocols for MPO with *N*-Acetyl-d-glucosamine; Figure S8: Percentage inhibition of MPO peroxidation activity with compounds **2a**, **2e**, **2f**, **2h**, Indomethacin and 5-ASA; Figures S9–S38: ^1^H, ^13^C NMR and IR spectra of compounds **2a**–**j**. Table S1: C2-C1-C7-C8 torsion angles of compounds **2a**–**j** in their neutral, radical and cation radical forms: Table S2: Cell viability assays; Procedures of acetamidocoumarins synthesis **1a**–**j**. MO calculations at B3LYP/6-31 + G(d,p), energies, imaginary frequencies and Z-matrix of **2a**–**j** in neutral, radical and cation radical forms.
