# Supplementary material for: Myeloperoxidase Inhibitory and Antioxidant Activities of (E)-2-Hydroxy-α-aminocinnamic Acids Obtained through Microwave-Assisted Synthesis
Source: Pharmaceuticals (Basel). 2021 May 27;14(6):513. doi: 10.3390/ph14060513 (PMC8229396; doi:10.3390/ph14060513)
Supplement: Supplementary file 1 [file pharmaceuticals-14-00513-s001.zip › pharmaceuticals-1200864-supplementary.pdf]

SUPPLEMENTARY MATERIAL

Myeloperoxidase inhibitory and antioxidant activities of (*E*)-2-hydroxy-α-aminocinnamic acids obtained through microwave assisted synthesis

Astrid M. Rivera-Antonio[1,2], Martha C. Rosales-Hernández[2], Irving Balbuena-Rebolledo[1,3], José Martín Santiago-Quintana[1,2], Jessica Elena Mendieta-Wejebe[2], José Correa-Basurto[3], Juan Benjamín García-Vázquez[3], Efrén V. García-Báez[1], Itzia I. Padilla-Martínez[1*]

[1] Laboratorio de Química Supramolecular y Nanociencias, Unidad Profesional Interdisciplinaria de Biotecnología, Instituto Politécnico Nacional, Avenida Acueducto s/n, Barrio la Laguna Ticomán, Ciudad de México 07340, Mexico, ipadillamar@ipn.mx

[2] Laboratorio de Biofísica y Biocatálisis, Sección de Estudios de Posgrado e Investigación, Escuela Superior de Medicina, Instituto Politécnico Nacional, Plan de San Luis y Salvador Díaz Mirón s/n, Casco de Santo Tomas, Ciudad de México 11340, Mexico.

[3] Laboratorio de Diseño y Desarrollo de Nuevos Fármacos e Innovación Biotecnológica, Escuela Superior de Medicina, Instituto Politécnico Nacional, Plan de San Luis y Díaz Mirón, s/n, Col. Casco de Santo Tomas, 11340, Ciudad de México, Mexico.

* Correspondence: ipadillamar@ipn.mx

List of contents:

1. Reaction monitoring by ^{1}H-NMR: Figures S1-2.
2. Antioxidant tests: Figures S3-6.
3. Theoretical calculations: Table S1 and Figure S7.
4. MPO and cell viability results: Figure S8 and Table S2.
5. Procedures of acetamidocoumarins synthesis **1a-j**
6. ^{1}H, ^{13}C NMR and IR spectra of **2a-j**: Figures S19-48.
7. MO calculations at B3LYP/6-31+G(d,p). Energies and imaginary frequencies of **2a-j**.

8. Z-matrix of **2a-j** in neutral, radical and cation radical forms.

1. Reaction monitoring by ¹H-NMR.

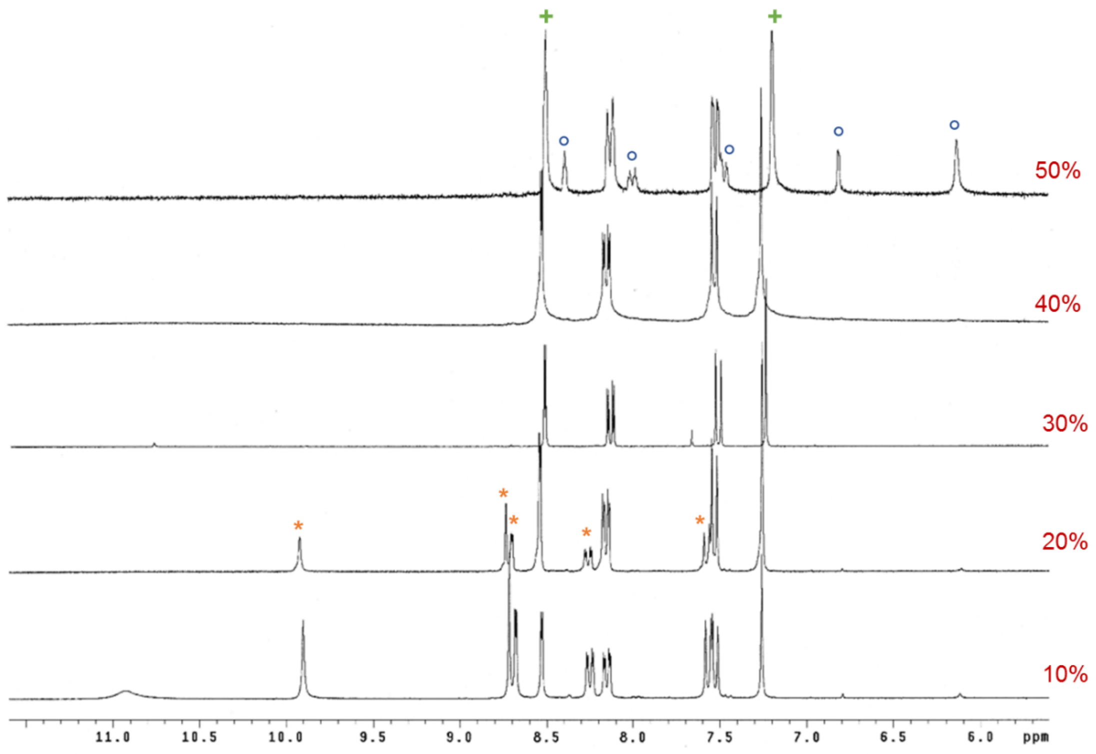

Figure S1. ¹H-NMR spectra (DMSO-d6) showing the effect of the H_2SO_4 (%, v/v in water) concentration on the conversion of the starting compound **1i** (*) to **2i** (+) and finally to the aminocoumarin (°), at a reaction temperature of 120 °C and 15 min of reaction time.

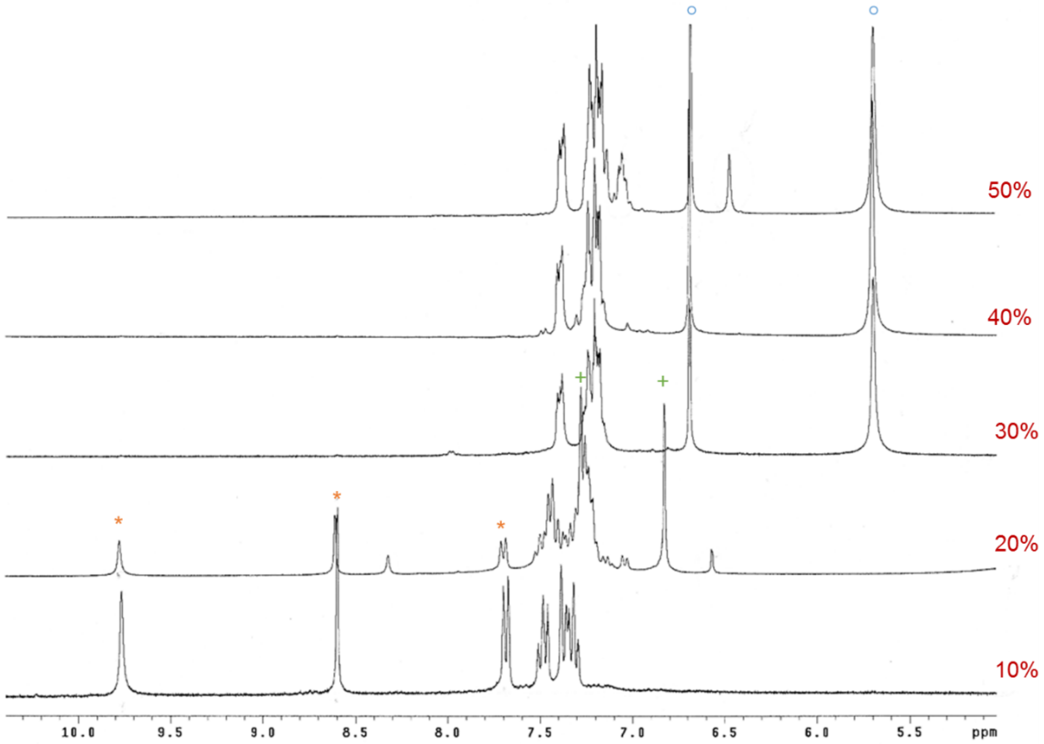

Figure S2. ^{1}H-NMR spectra (DMSO-d6) showing the effect of the H_2SO_4 (%, v/v in water) concentration on the conversion of the starting compound **1a** (*) to **2a** (+) and finally to the aminocoumarin (°), at a reaction temperature of 120 ° C and 15 min of reaction time.

2. Antioxidant tests

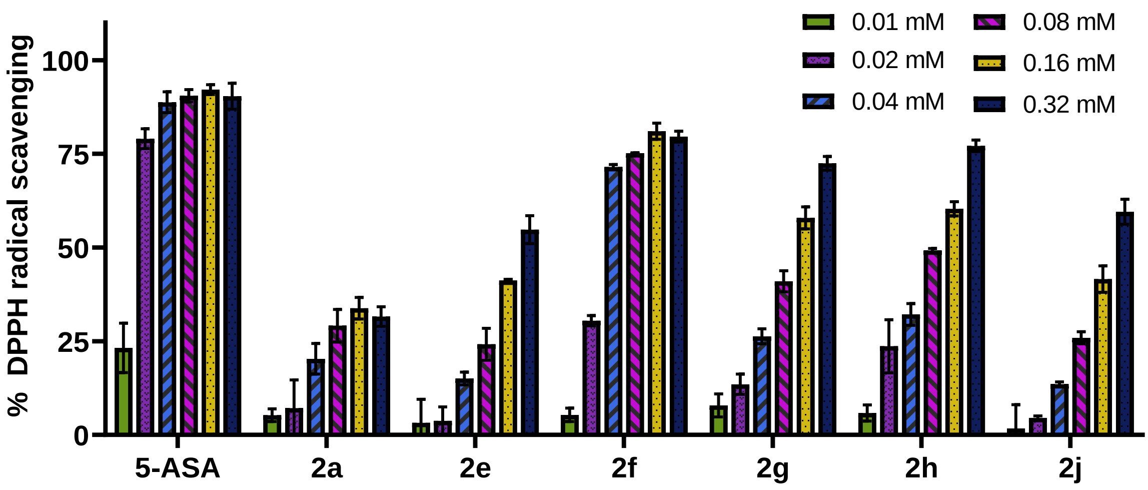

Figure S3. Percentage of DPPH RSA with compounds **2a**, **2e**, **2f**, **2g**, **2h** and **2j**.

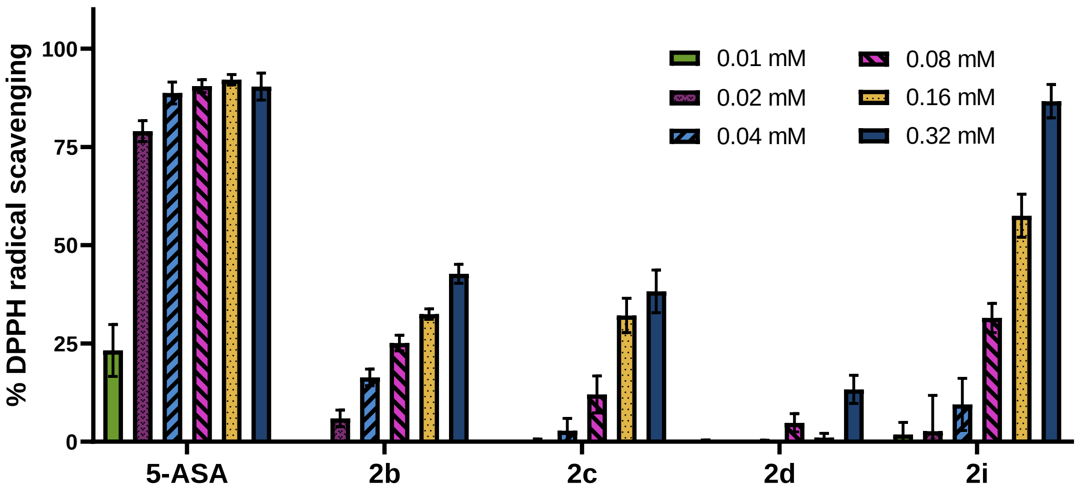

Figure S4. Percentage of DPPH RSA with compounds **2b**, **2c**, **2d**, **2i**.

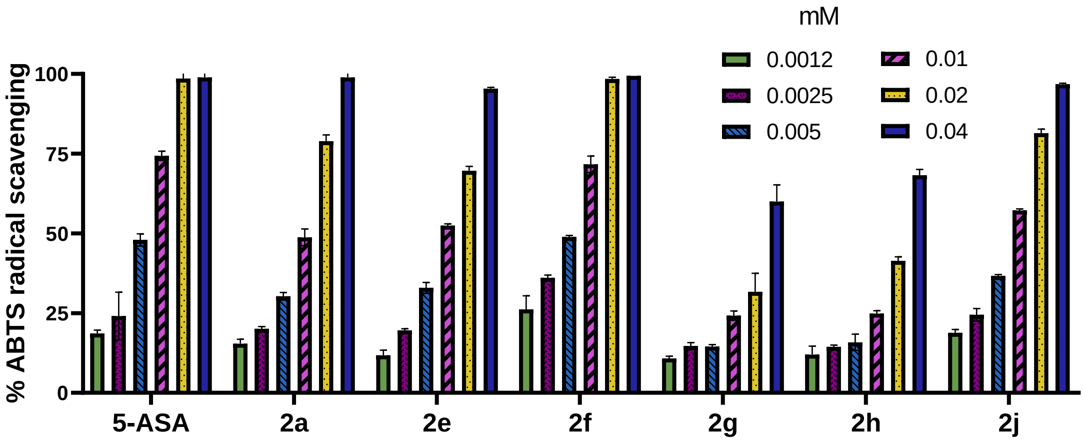

Figure S5. Percentage of ABTS RSA with compounds **2a**, **2e**, **2f**, **2g**, **2h** and **2j**.

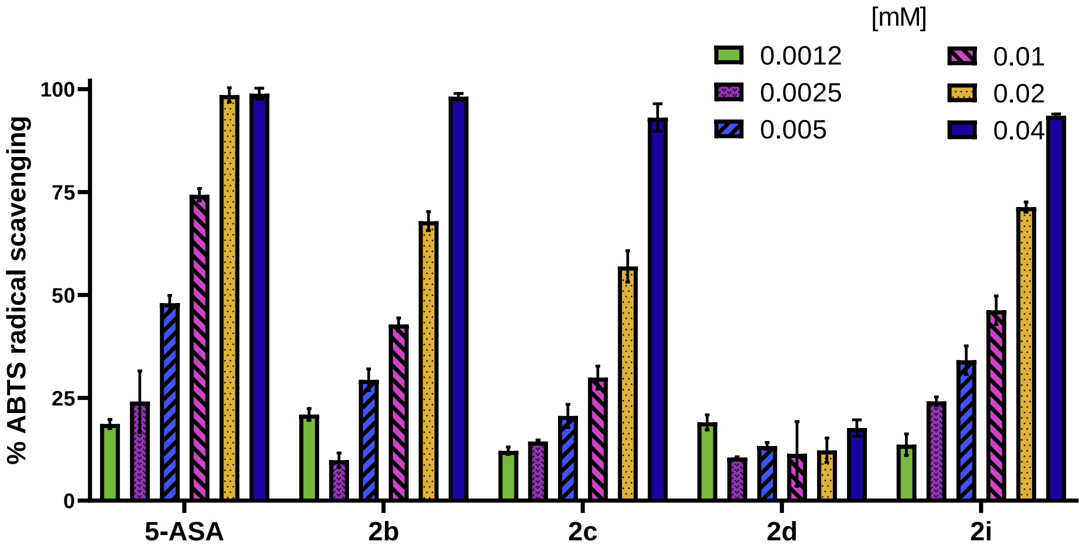

Figure S6. Percentage of ABTS RSA with compounds **2b**, **2c**, **2d**, **2i**.

3. Theoretical calculations

Table S1. C2-C1-C7-C8 torsion angle in degrees of compounds **2a-j** in their neutral, radical and cation radical forms.

Compound	Neutral	Radical	Cation
**2a**	145.24	22.23	24.06
**2b**	144.91	23.81	29.39
**2c**	144.5	23.92	28.85
**2d**	145.79	23.99	25.55

**2e**	146.14	25.97	24.23
**2f**	146.41	20.74<br>26.76	23.92
**2g**	145.47	25.92	29.03
**2h**	145.64	26.56<br>141.34	27.90
**2i**	52.57	20.58	27.11
**2j**	143.43	28.76	33.69

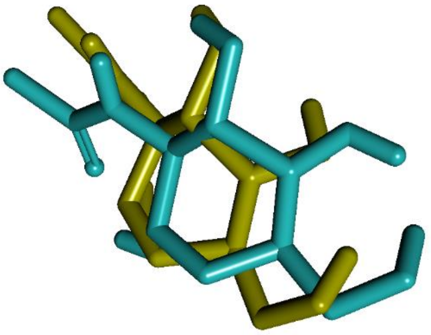

Figure S7. Validation of the molecular docking protocols for the Myeloperoxidase (PDB code: 1DNU) with N-Acetyl-D-glucosamine, RMSD: 1.194 Å.

4. MPO and cell viability results

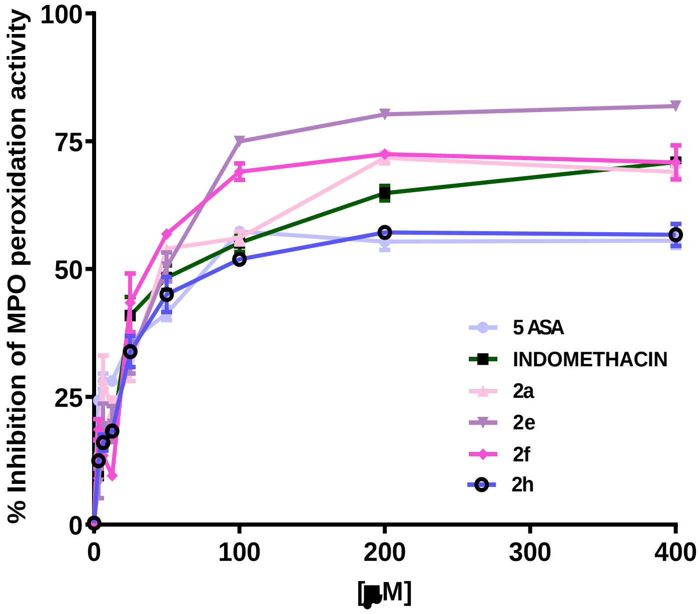

Figure S8. Percentage inhibition of MPO peroxidation activity with compounds **2a**, **2e**, **2f**, **2h**, Indomethacin and 5-ASA.

Table S2. Cell viability as the percent of MTT reduction in NIH/3T3 cells after treatment with compounds derived from cinnamic acid at 12.5 -200 µM mean and ± SEM.

Concentration µM	5-ASA	2a	2b	2c	2d	2e	2f	2g	2h	2i
**12.5**	92.5 ± 3.1	100.9 ± 1.9	97.6 ± 1.4	97.2 ± 1.9	98.0 ± 1.9	91.7 ± 2.1	90.0 ± 3.0	97.6 ± 2.0	83.3 ± 2.3	96.8 ± 2.6
**25**	92.3 ± 2.7	97.9 ± 2.4	95.0 ± 1.6	96.4 ± 2.1	96.8 ± 1.8	89.3 ± 2.2	90.5 ± 3.5	95.4 ± 4.9	90.9 ± 3.5	94.4 ± 1.9
**50**	91.1 ±3.8	95.6 ± 1.7	94.0 ± 2.1	89.4 ± 2.6	94.5 ± 1.7	91.0 ± 2.6	93.7 ± 2.6	94.8 ± 2.9	90.9 ± 3.4	94.2 ± 2.3
**100**	83.2 ± 2.8	94.2 ± 2.7	91.9 ± 1.0	88.1 ± 2.3	94.7 ± 2.7	91.9 ± 1.8	91.9 ± 2.9	87.8 ± 2.7	91.5 ± 2.9	93.6 ± 2.4
**200**	83.9 ± 2.1	91.7 ± 3.5	80.7 ± 1.5	91.0 ± 2.3	87.3 ± 2.3	94.1 ± 1.2	86.9 ± 3.2	84.6 ± 2.1	90.0 ± 2.4	93.0 ± 2.1

5. Procedures of acetamidocoumarins **1a-j** synthesis.

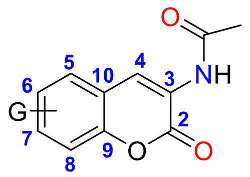

1

a 6-H, **b** 6-Br, **c** 6-Cl, **d** 6-F, **e** 8-EtO, **f** 7-OAc,
g 6-MeO, **h** 6-AcO, **i** 6-NO_2, **j** 6-NH_2

N-(2-oxo-2*H*-chromen-3-yl) acetamide (**1a**). Into a 100 mL beaker flask were placed 1.8 mL of salicylaldehyde (16 mmol), 1.31 g of NaOAc (16.0 mmol), 1.20 g of glycine (16.0 mmol) and 20 mL of acetic anhydride at reflux for 26 h. At the end of the reaction, 30 mL of cold distilled water was added to the reaction mixture once cooled to room temperature. The resulting solid was separated by filtration and washed with 10 mL of distilled AcOEt, after drying, 3.03 g of a brown solid was obtained in 80% yield. Rf: 0.66 Hexane / Acetate 1:1. ^{1}H NMR (DMSO-*d6*): δ 9.71 (s, 1H, NH), 8.55 (s, 1H, H4), 7.63 (d, 1H, ^3J = 7, H5), 7.43 (t, 1H, ^3J = 7, H6), 7.29 (m, 2H, H7-8), 2.10 (s, 3H, Me).

N-(6-bromo-2-oxo-2*H*-chromen-3-yl) acetamide (**1b**). It was synthesized and isolated as indicated for compound **1a**, starting from 2.00 g of 5-bromo-salicylaldehyde (8.00 mmol), 0.816 g of NaOAc (8.00 mmol) and 0.746 g of glycine (8.00 mmol) to obtain 2.17 g of a brown solid in 86% yield. Rf: 0.75 Hexane / Acetate 1:1. ^{1}H NMR (DMSO-*d6*): δ 9.81 (s, 1H, NH), 8.56 (s, 1H, H4), 7.97 (d, 1H, ^4J = 2.3, H5), 7.61 (dd, 1H, ^3J = 8.8, ^4J = 2.3, H7), 7.33 (d, 1H, ^3J = 8.8, H8), 2.15 (s, 3H, Me).

N-(6-cloro-2-oxo-2*H*-cromen-3-il) acetamide (**1c**). It was synthesized and isolated as indicated for compound **1a**, starting from 2.00 g of 5-chloro-salicylaldehyde (12.7 mmol), 1.04 g of NaOAc (12.7 mmol) and 0.958 g of glycine (12.7 mmol) to obtain 2.92 g of a brown solid in 85% yield. Rf: 0.81 Hexane / Acetate 1:1. ^{1}H NMR (DMSO-*d6*): δ 8.60 (s, 1H, H4), 8.10 (a, 1H, NH), 7.48 (d, 1H, ^4J = 2.4, H5), 7.39 (dd, 1H, ^3J = 8.8, ^4J = 2.4, H7), 7.22 (dd, 1H, ^3J = 8.8, ^4J = 2.4, H8), 2.25 (s, 3H, Me).

N-(6-fluoro-2-oxo-2*H*-chromen-3-yl) acetamide (**1d**). It was synthesized and isolated as indicated for compound **1a**, starting from 0.500 g of 5-fluoro-salicylaldehyde (3.50 mmol), 0.292 g of NaOAc (3.50 mmol), 0.267 g of glycine (3.5 mmol) and 10 mL of acetic anhydride to obtain 0.740 g of a brown solid in 83% yield. Rf: 0.83 Hexane / Acetate 1:1. ^{1}H NMR (DMSO-*d6*): δ 9.79 (s, 1H, NH), 8.57 (s, 1H, H4), 7.60 (dd, 1H, $^3J_{H-F}$ = 8.8, ^4J = 3.0, H5), 7.38 (m, 1H, H8), 7.30 (dd, 1H, $^3J_{H-F}$ = 8.8, ^3J = 8.2, H7), 2.14 (s, 3H, Me).

N-(8-ethoxy-2-oxo-2*H*-chromen-3-yl) acetamide (**1e**). It was synthesized and isolated as indicated for compound **1a**, starting from 2.00 g of 3-ethoxy-salicylaldehyde (12.0 mmol), 0.98 g of NaOAc (12.0 mmol) and 0.897 g of glycine (12.0 mmol) to obtain 2.63 g of a brown solid in 78% yield. Rf: 0.69 Hexane / Acetate 1:1. ^{1}H NMR (DMSO-*d6*): δ 8.54 (s, 1H, H4), 8.10 (a, 1H, NH), 7.20 (t, 1H, ^3J = 7.9, H6), 7.11 (dd, 1H, ^3J = 7.6, ^4J = 1.0, H5), 6.99 (dd, 1H, ^3J = 7.6, ^4J = 1.0, H7), 4.18 (c, 2H, ^3J = 7.0, CH_2) 2.24 (s, 3H, MeCO), 1.50 (t, 3H, ^3J = 7.0, Me).

3-Acetamido-2-oxo-2*H*-chromen-7-yl acetate (**1f**). It was synthesized and isolated as indicated for compound **1a**, starting from 2.00 g of 4-hydroxy-salicylaldehyde (14.0 mmol), 1.04 g of NaOAc (14.0 mmol) and 0.958 g of glycine (14.0 mmol) to obtain 2.72 g of a brown solid with 77% yield. Rf: 0.77 Hexane / Acetate 1:1. ^{1}H NMR (DMSO-*d6*): δ 9.76 (s, 1H, NH), 8.60 (s, 1H, H4), 7.73 (d, ^3J = 8.82, H5), 7.26 (d, 1H, ^4J = 2.4, H8), 7.13 (dd, 1H, ^3J = 8.6, ^4J = 2, H6), 2.29 (s, 3H, MeO), 2.16 (s, 3H, MeCO).

N-(6-methoxy-2-oxo-2*H*-chromen-3-yl) acetamide (**1g**). It was synthesized and isolated as indicated for compound **1a**, starting from 1.64 mL 5-methoxy-salicylaldehyde (13.0 mmol), 1.08 g of NaOAc (13.0 mmol) and 0.987 g of glycine (13.0 mmol) to obtain 2.77 g of a brown solid in 80% yield. Rf: 0.65 Hexane / Acetate 1:1. ^{1}H NMR (DMSO-*d6*): δ 8.63 (s, 1H, H4), 8.10 (s, 1H, NH), 7.23 (d, 1H, ^3J = 8.8, H7), 6.95 (d, 1H, ^3J = 8.8, H8), 6.94 (d, 1H, ^4J = 3, H5), 3.84 (s, 3H, MeO), 2.24 (s, 3H, MeCO).

3-Acetamido-2-oxo-2*H*-chromen-6-yl acetate (**1h**). It was synthesized and isolated as indicated for compound **1a**, starting from 2.00 g of 5-hydroxy-salicylaldehyde (14.0 mmol), 1.18 g of NaOAc (21 mmol) and 1.08 g of glycine (14.0 mmol) to obtain 1.96 g of a brown solid in 62% yield. Rf: 0.78 Hexane / Acetate 1:1. ^{1}H NMR (DMSO-*d6*): δ 9.80 (s, 1H, NH), 8.57 (s, 1H, H4), 7.50 (d, 1H, ^3J = 2.9, H5), 7.42 (d, 1H, ^3J = 8.8, H8), 7.40 (dd, 1H, ^4J = 2.4, ^3J = 8.8, H7), 2.286 (s, 3H, OAc), 2.17 (s, 3H, NAc).

N-(6-nitro-2-oxo-2*H*-chromen-3-yl) acetamide (**1i**). It was synthesized and isolated as indicated for compound 1a, starting from 2.00 g of 5-nitro-salicylaldehyde (12.0 mmol), 0.980 g of NaOAc (12.0 mmol) and 0.897 g of glycine

(12.0 mmol) to obtain 2.63 g of a brown solid in 78% yield. Rf: 0.70 Hexane / Acetate 1:1. ^{1}H NMR (DMSO-*d6*): 9.90 (s, 1H, NH), 8.73 (s, 1H, H4), 8.69 (d, 1H, ^{4}J = 2.4, H5), 8.26 (dd, 1H, ^{3}J = 9.2, ^{3}J = 2.5, H7), 7.58 (d, 1H, ^{3}J = 9.4, H8), 2.17 (s, 3H, Me).

N-(6-amino-2-oxo-2*H*-chromen-3-yl) acetamide (**1j**). Into a 1 L flat bottomed flask purged with nitrogen was placed 0.320 g of 10% Pd / C. In addition, in a beaker a solution was prepared with 1.21 g (32.9 mmol) of sodium borohydride and 20 mL of water, which was added dropwise to the flask for five minutes. A suspension was prepared with 3.20 g (12.9 mmol) of compound 1i in 600 mL of methanol and added to the above mixture dropwise over a period of 2 hours, then left stirring for 30 minutes and filtered with celite. The obtained solution was evaporated, the resulting honey was precipitated with 10 mL of cold water. The resulting solid was filtered under vacuum and dried at room temperature. 0.600 g of a yellow solid were obtained with a yield of 47%. Rf: 0.43 Hexane / Acetate 1:1. ^{1}H NMR (DMSO-*d6*): δ 9.68 (s, 1H, NH), 8.38 (s, 1H, H4), 7.13 (d, 1H, ^{3}J = 8.6, H7), 6.71 (dd, 1H, ^{3}J = 8.8, ^{4}J = 2.6, H8), 6.78 (d, 1H, ^{4}J = 2.6, H5), 5.25 (s, 2H, NH_2), 2.19 (s, 3H, Me).

Since compounds **1a-j** are known, their identity was confirmed with ^{1}H NMR.

6. ^{1}H, ^{13}C NMR and IR spectra of **2a-j**.

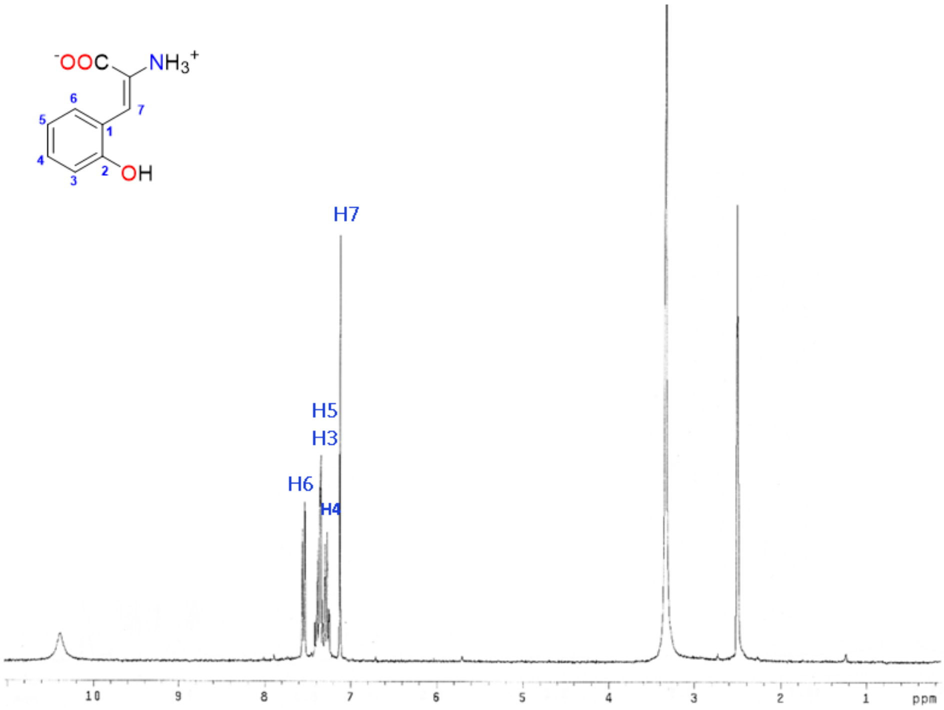

Figure S9. ^{1}H NMR spectrum of the compound **2a**, DMSO-*d6*.

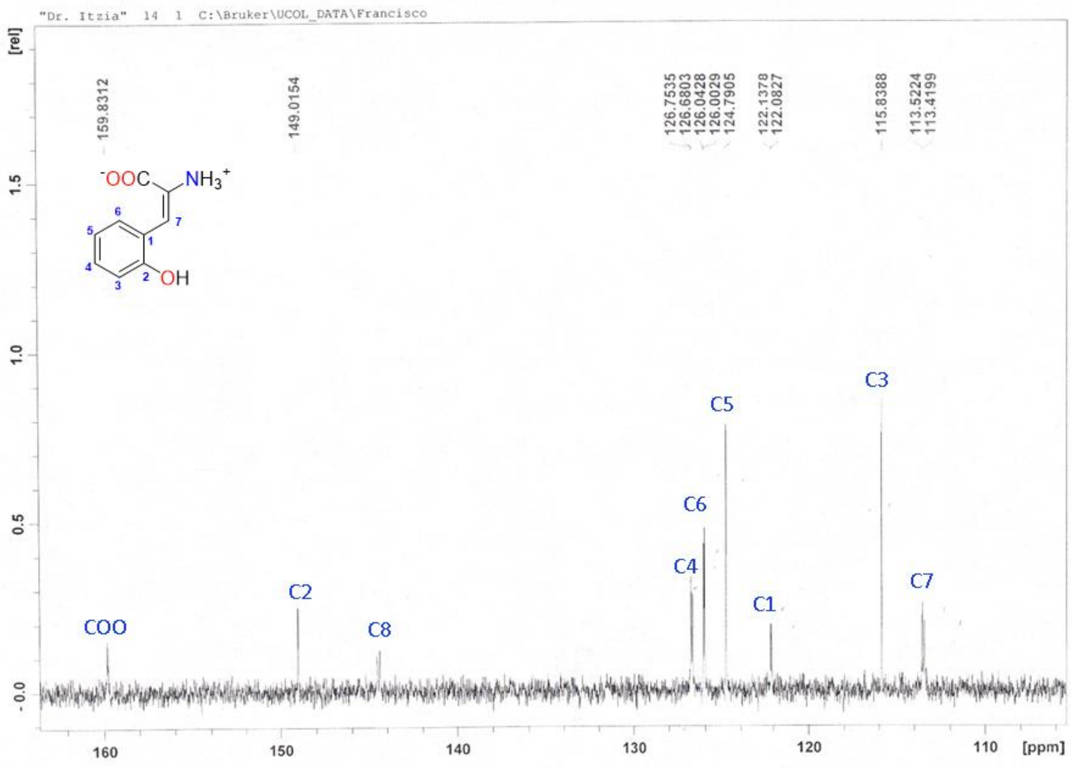

Figure S10. RMN ^{13}C NMR spectrum of the compound **2a**, DMSO-*d6*.

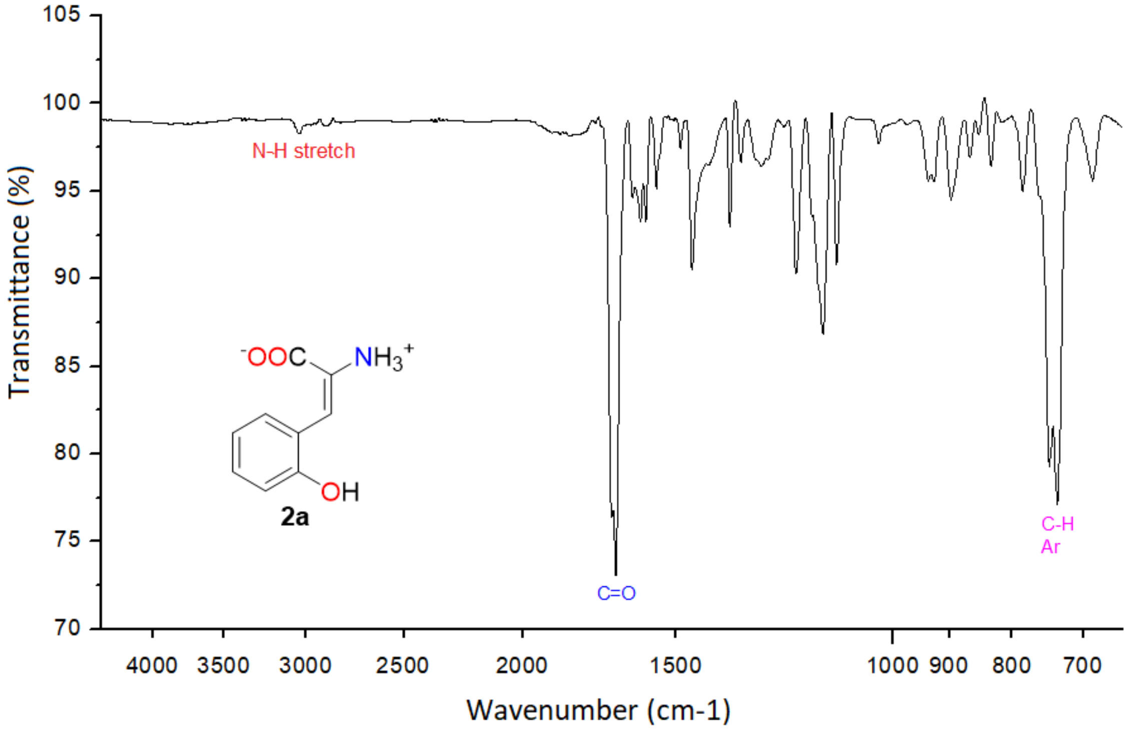

Figure S11. IR spectrum of compound **2a**.

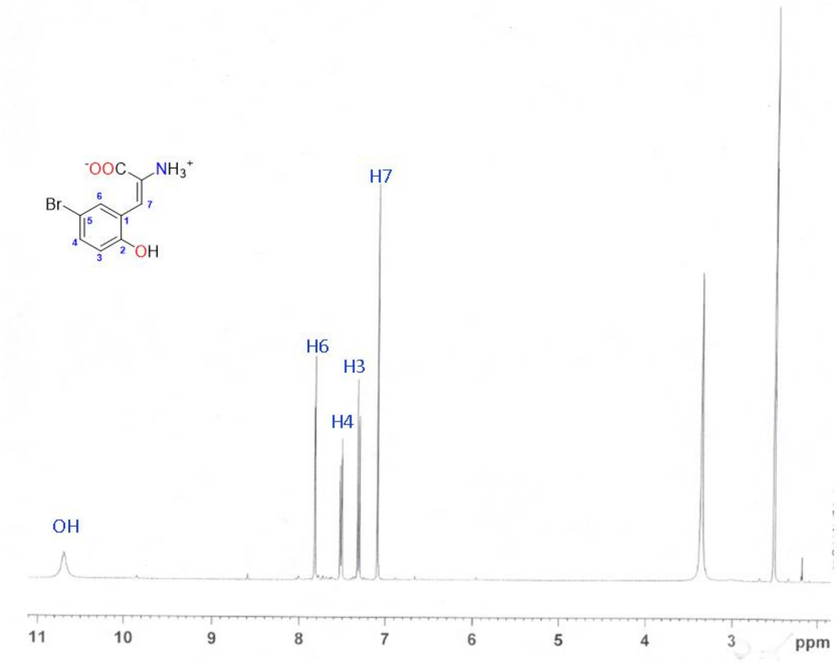

Figure S12. ^{1}H NMR spectrum of the compound **2b**, DMSO-*d6*.

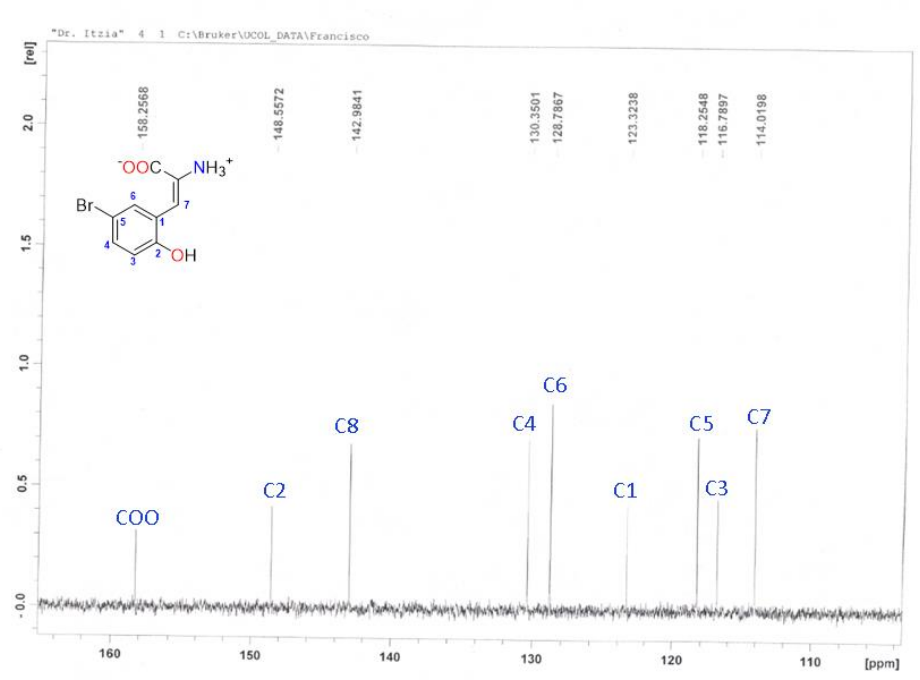

Figure S13. RMN ^{13}C NMR spectrum of the compound **2b**, DMSO-*d6*.

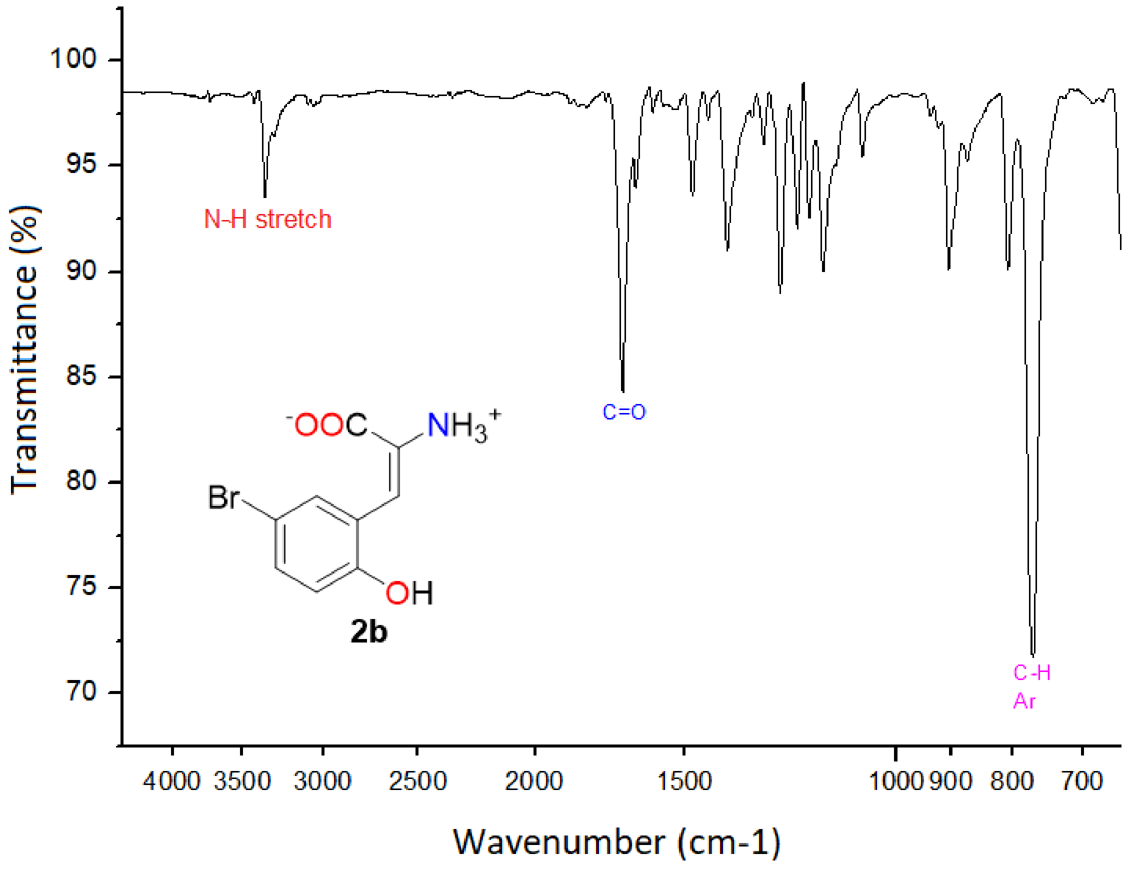

Figure S14. IR spectrum of compound **2b**.

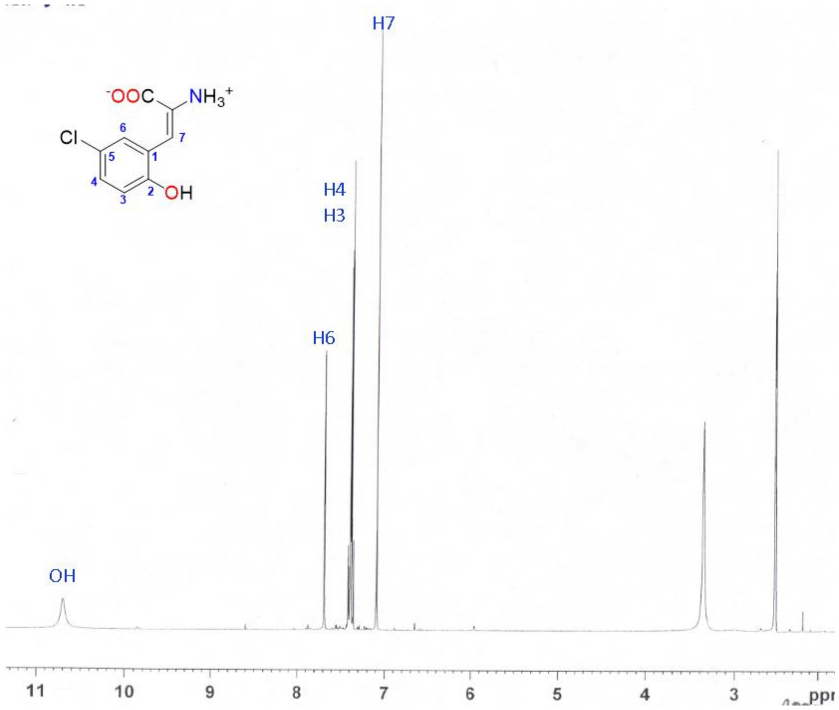

Figure S15. ^{1}H NMR spectrum of the compound **2c**, DMSO-*d*6.

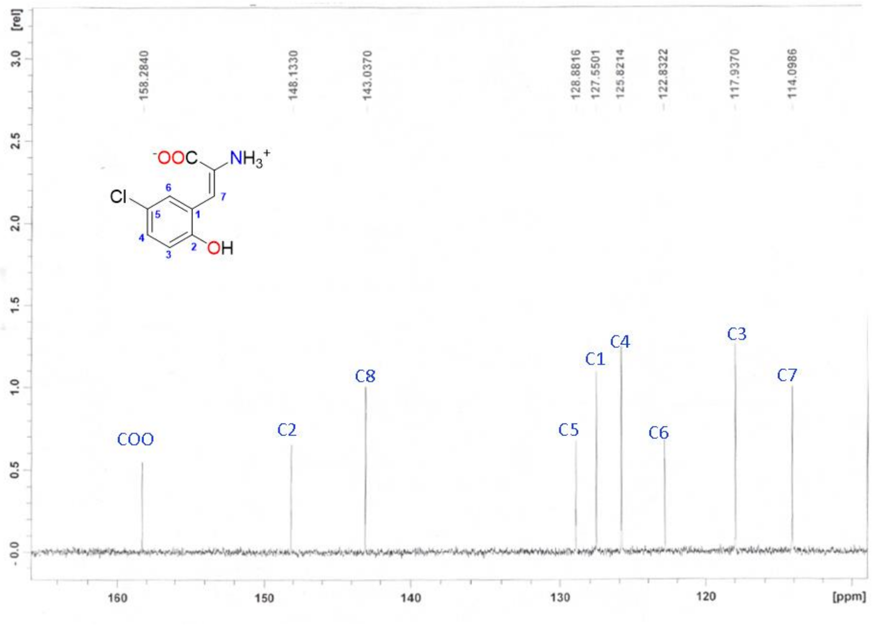

Figure S16. RMN ^{13}C NMR spectrum of the compound **2c**, DMSO-*d*6.

2c

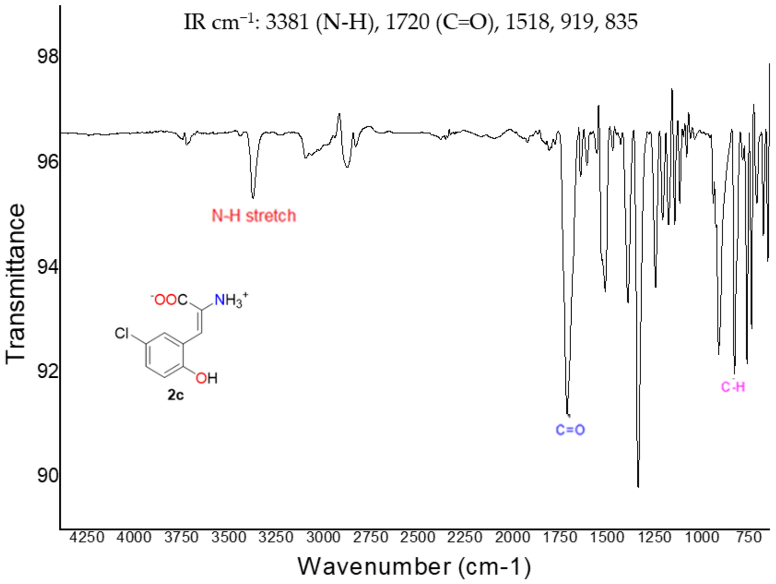

Figure S17. IR spectrum of compound **2c**.

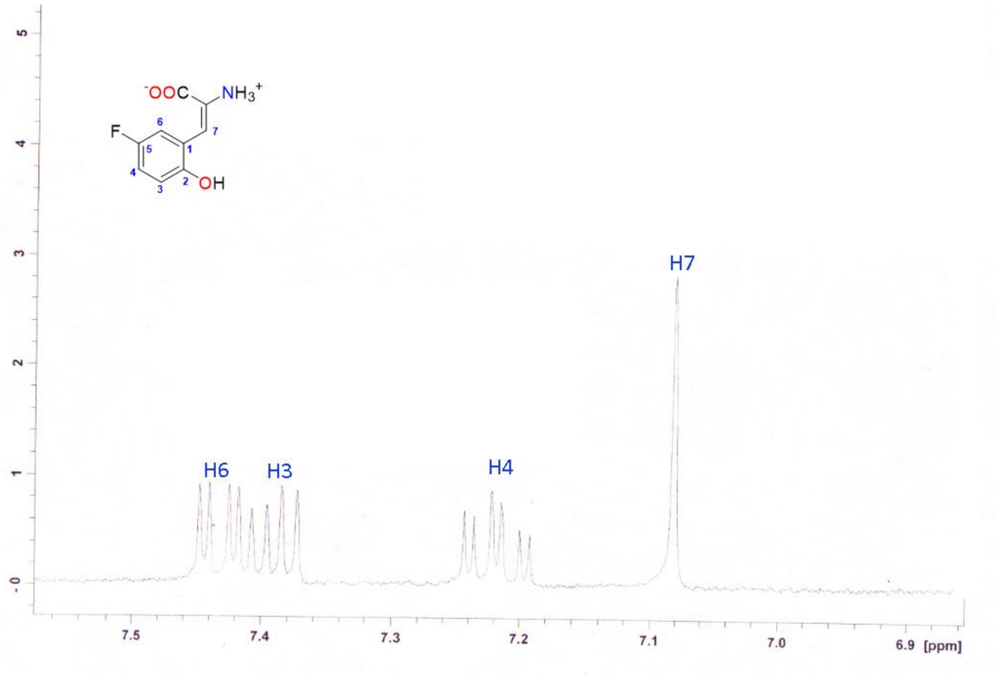

Figure S18.^{1}H NMR spectrum of the compound **2d**, DMSO-*d*6.

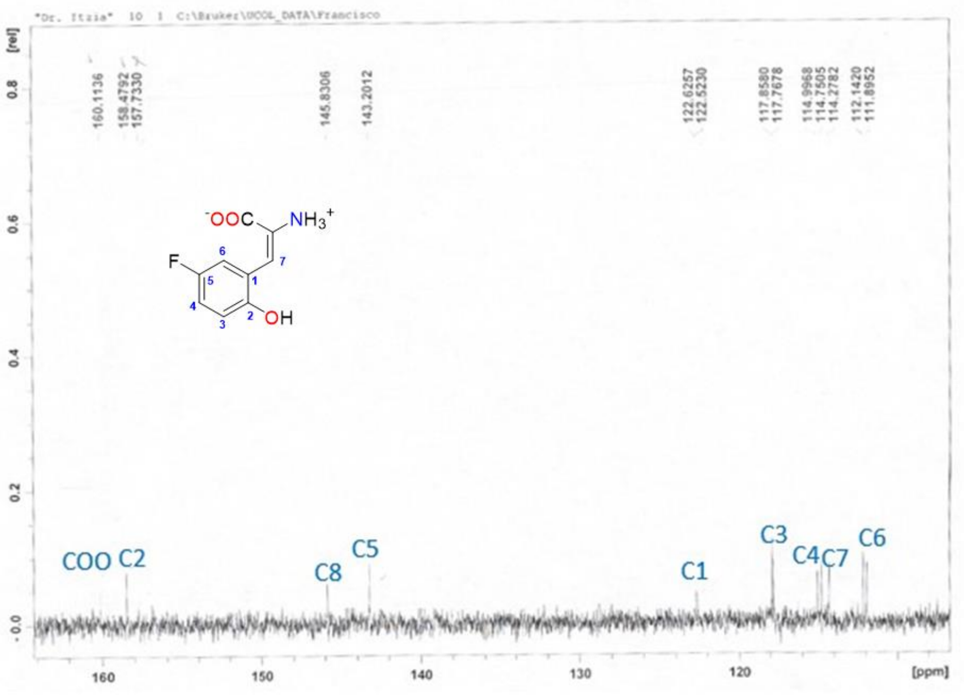

Figure S19. RMN ^{13}C NMR spectrum of the compound **2d**, DMSO-*d6*.

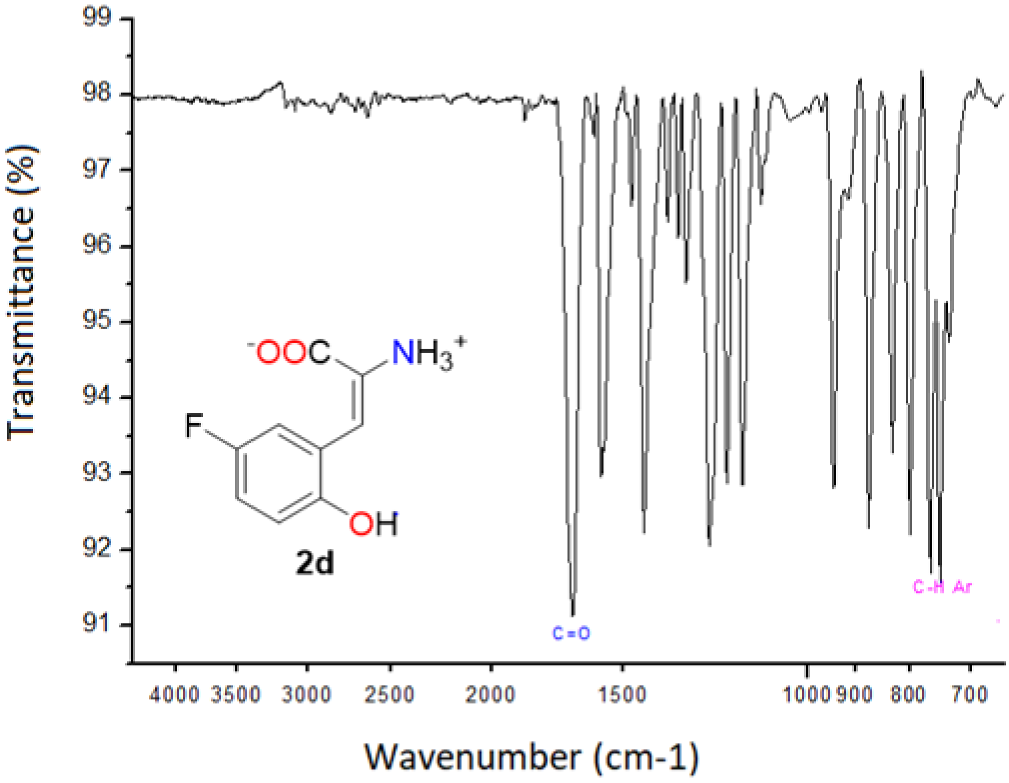

Figure S20. IR spectrum of compound **2d**.

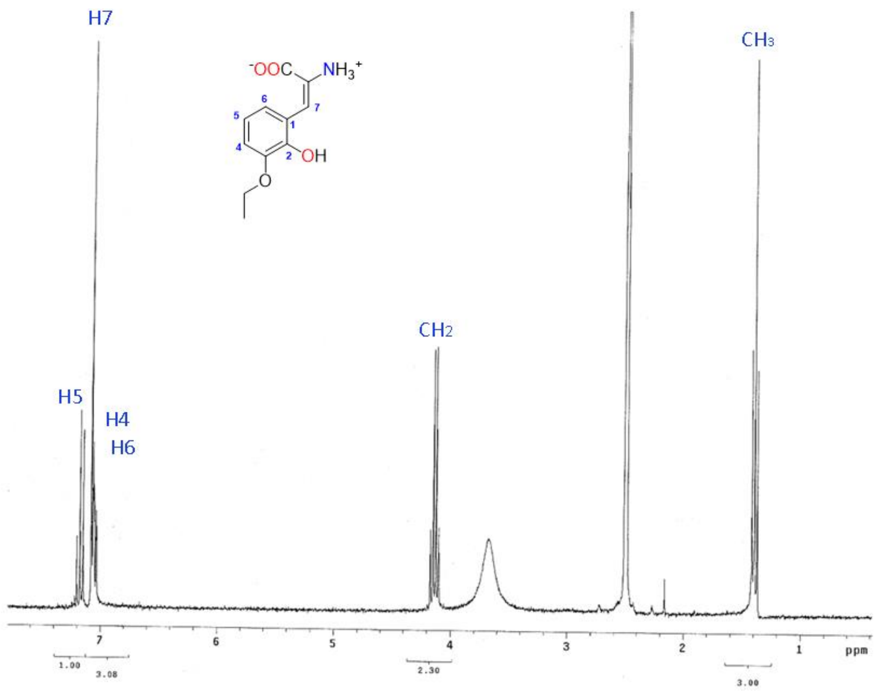

Figure S21. ^{1}H NMR spectrum of the compound **2e**, DMSO-*d*6.

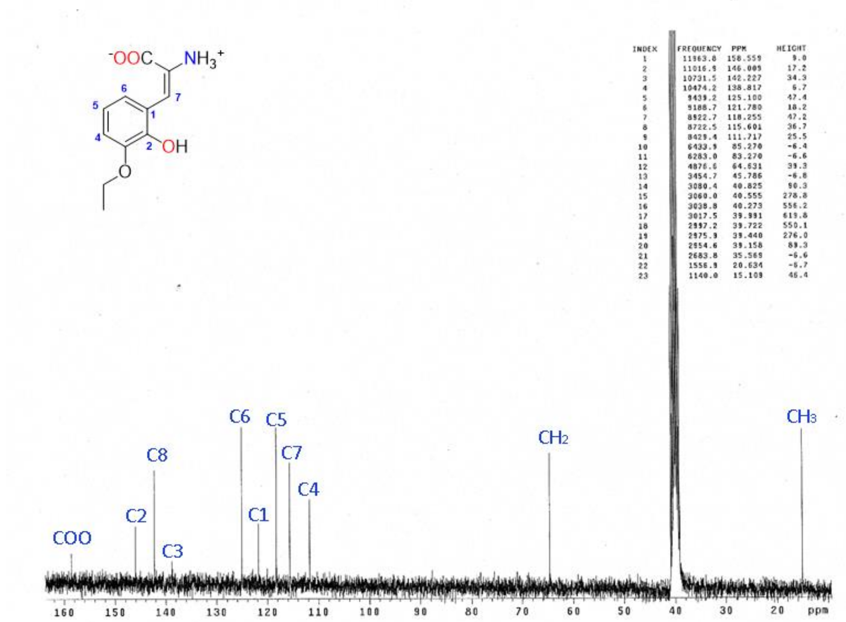

Figure S22. RMN ^{13}C NMR spectrum of the compound **2e**, DMSO-*d*6.

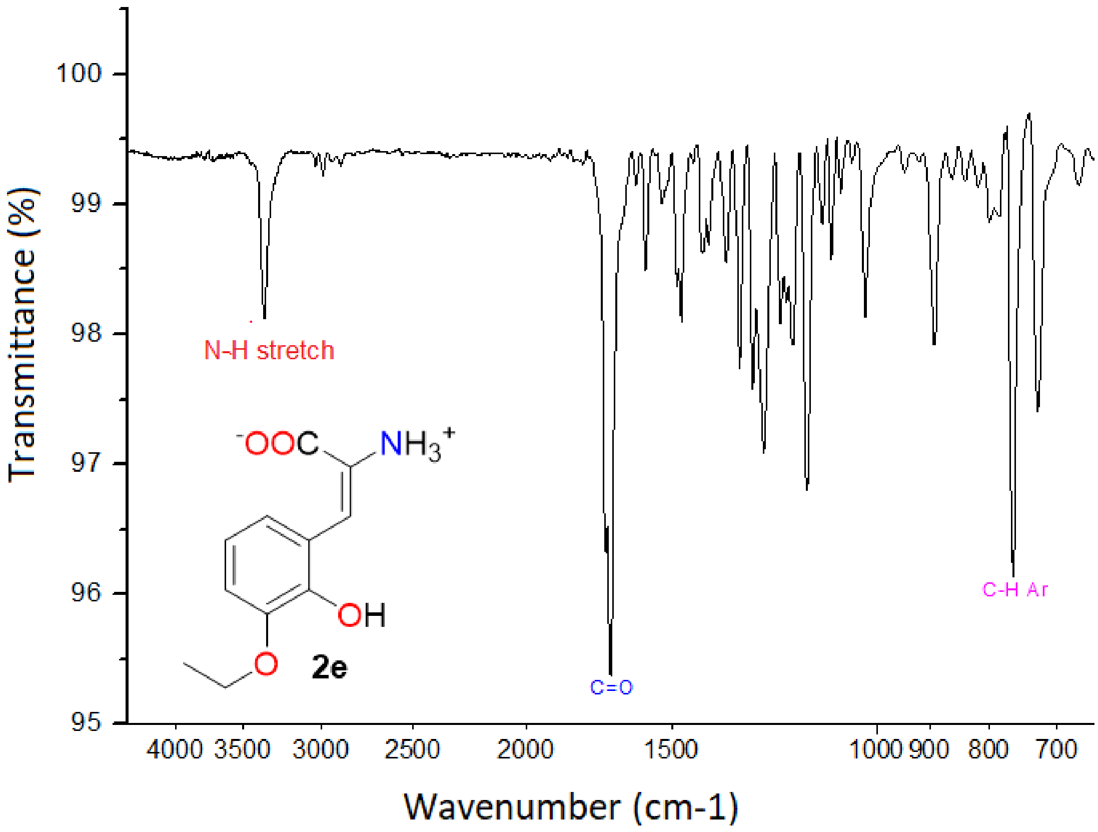

Figure S23. IR spectrum of compound **2e**.

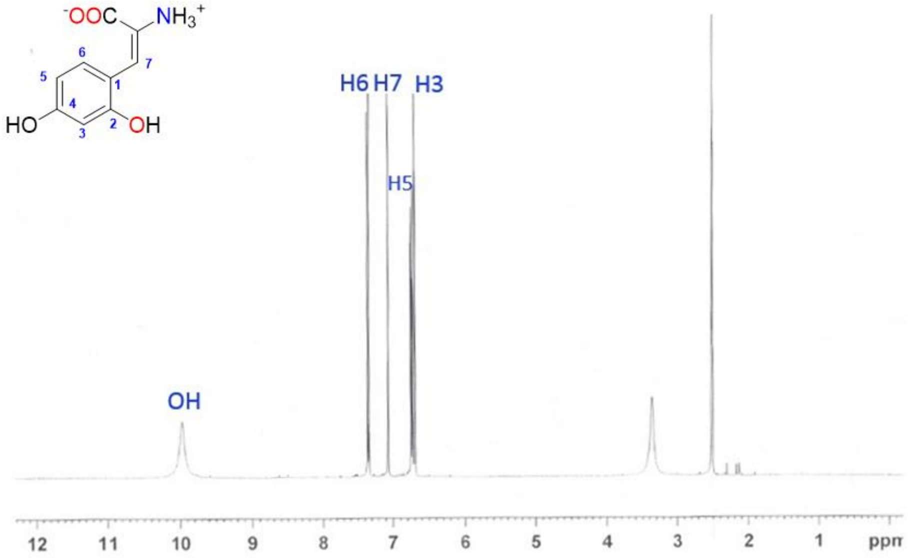

Figure S24. ^{1}H NMR spectrum of the compound **2f**, DMSO-*d*6.

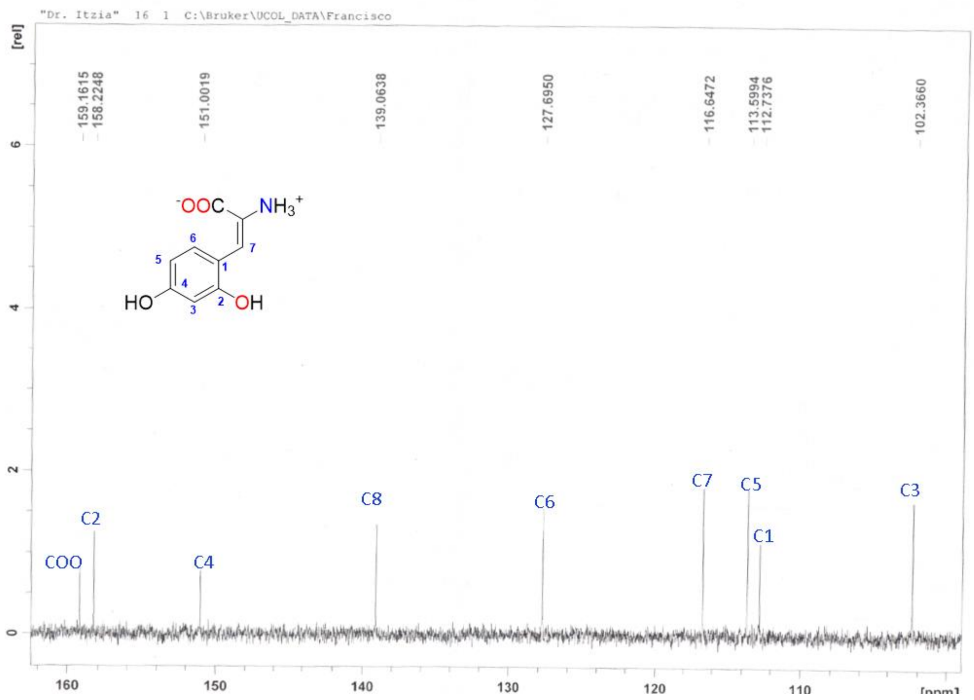

Figure S25. RMN ^{13}C NMR spectrum of the compound **2f**, DMSO-*d*6.

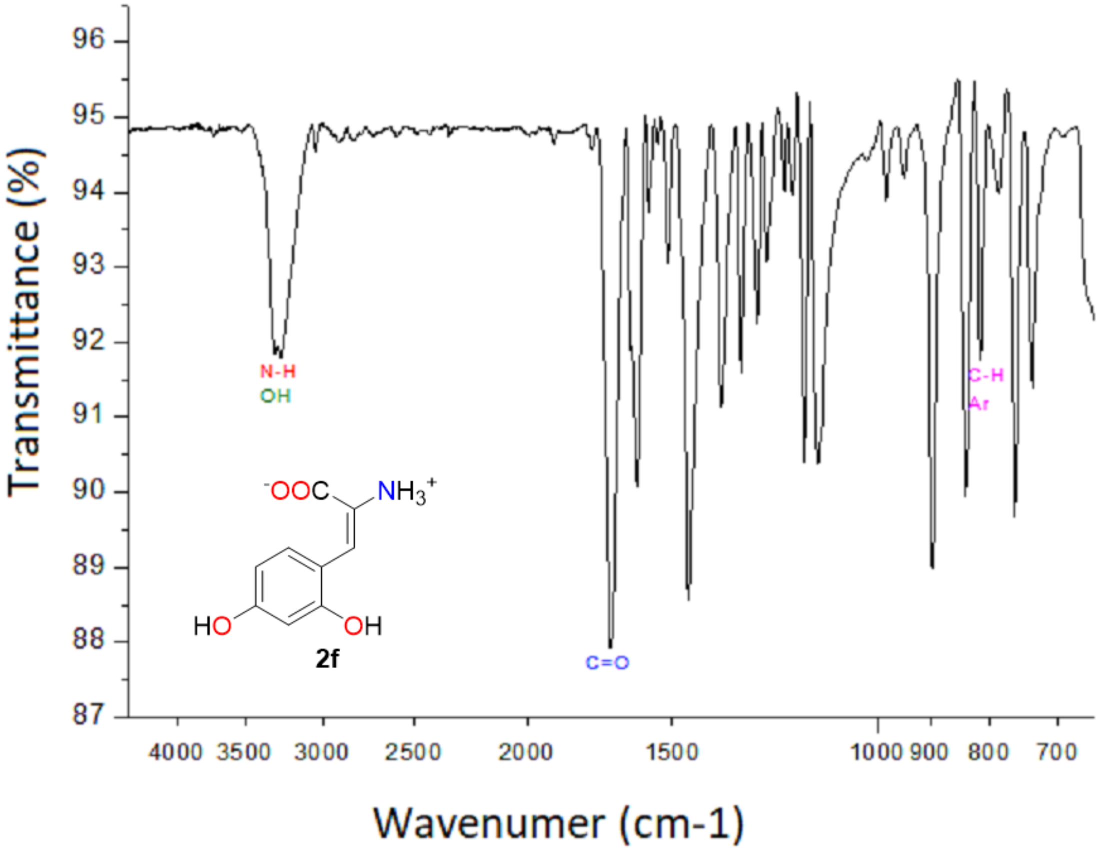

Figure S26. IR spectrum of compound **2f**.

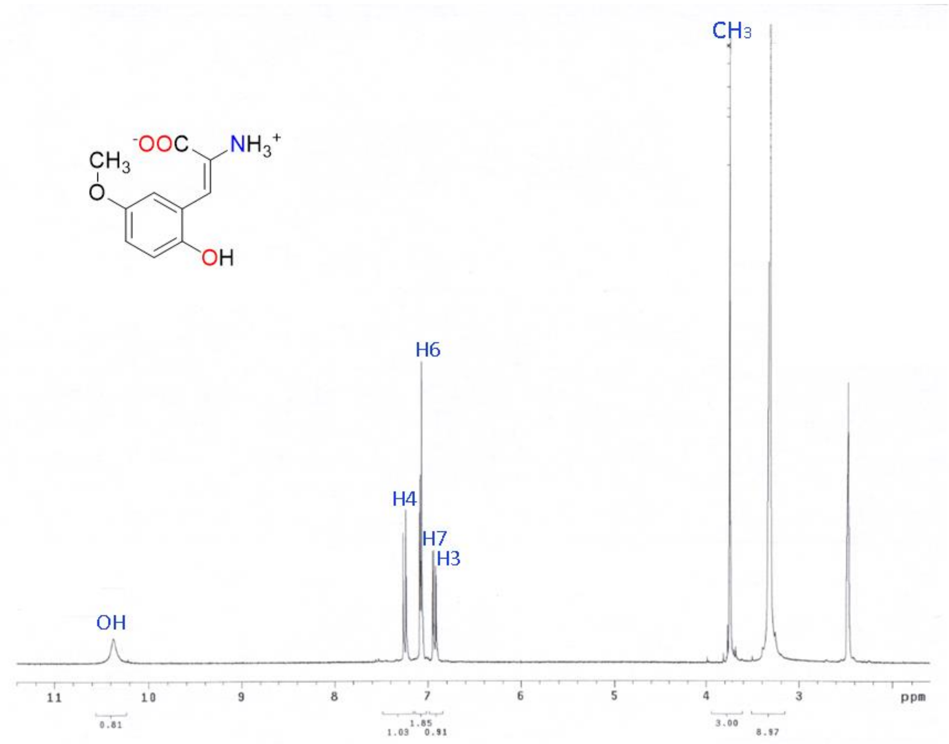

Figure S27. ^{1}H NMR spectrum of the compound **2g**, DMSO-*d*6.

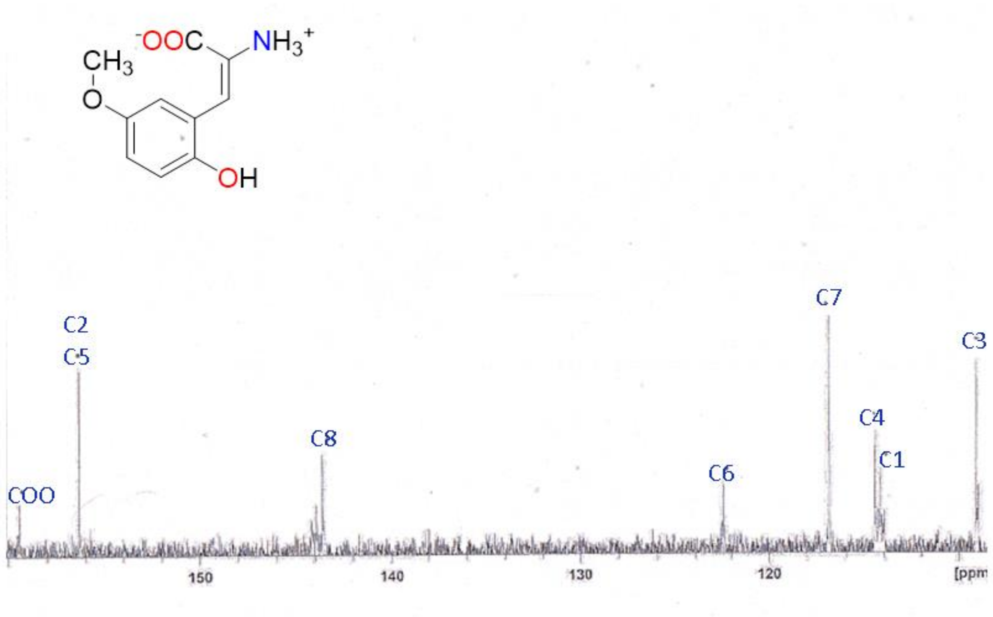

Figure S28. RMN ^{13}C NMR spectrum of the compound **2g**, DMSO-*d*6.

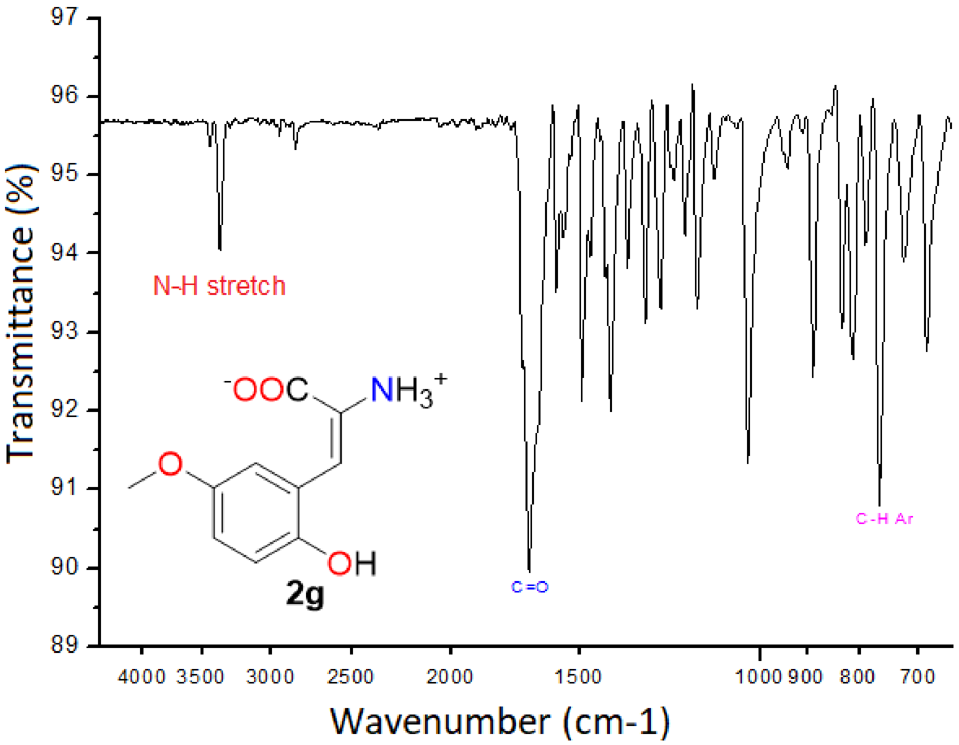

Figure S29. IR spectrum of compound **2g**.

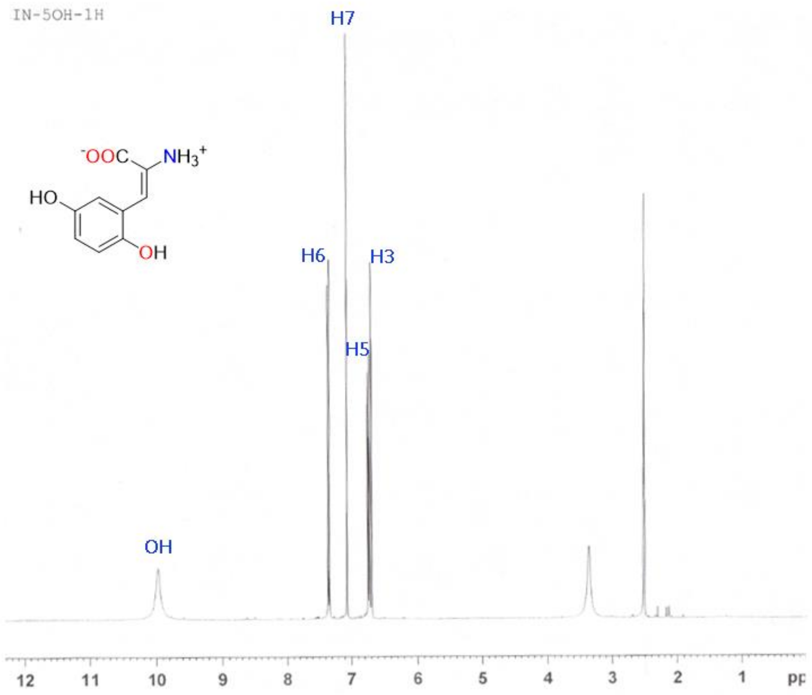

Figure S30. ^{1}H NMR spectrum of the compound **2h**, DMSO-*d6*.

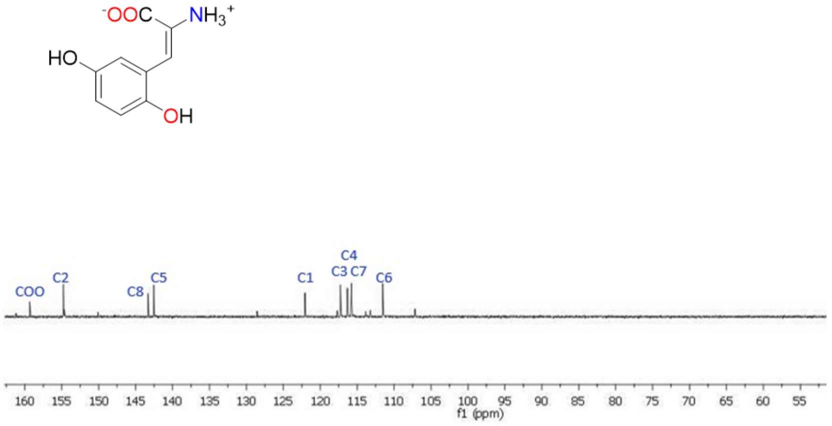

Figure S31. RMN ^{13}C NMR spectrum of the compound **2h**, DMSO-*d6*.

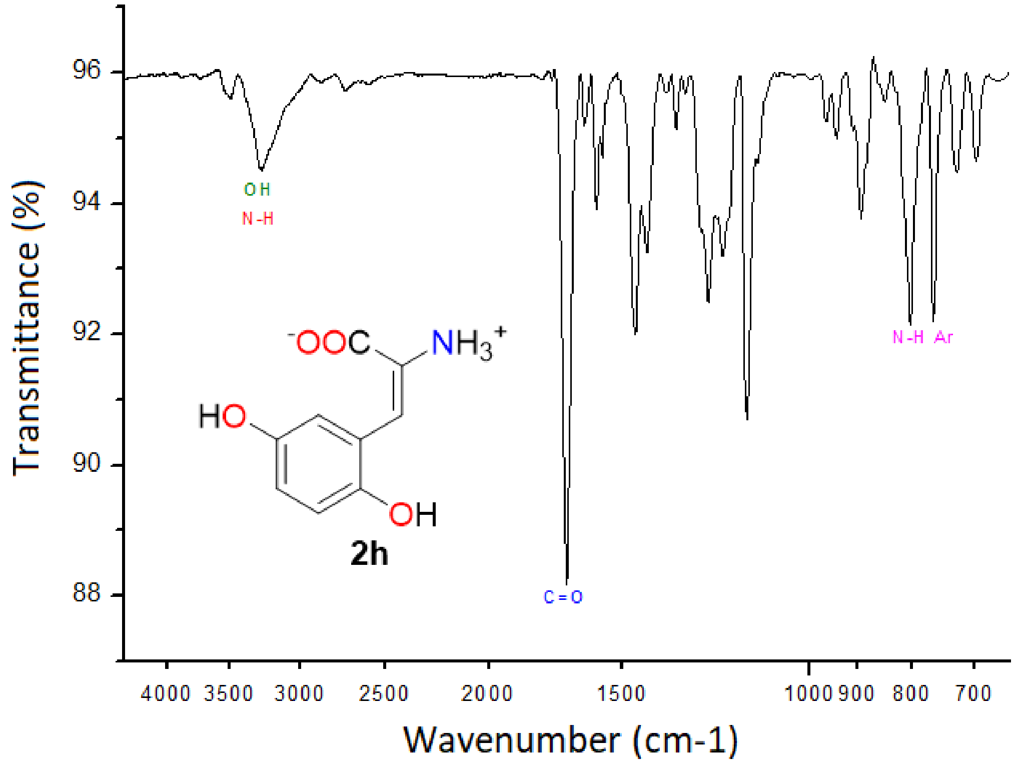

Figure S32. IR spectrum of compound **2h**

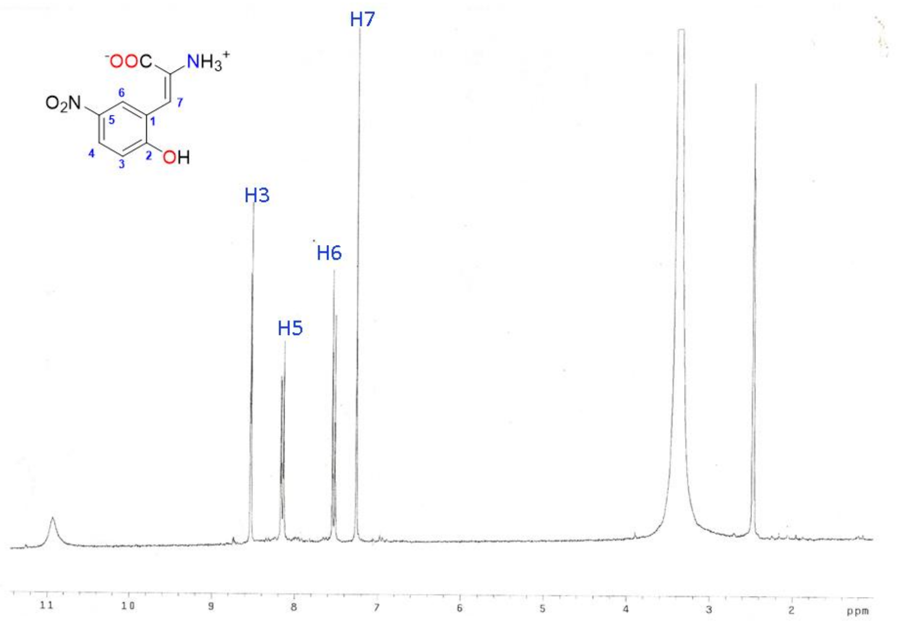

Figure S33. ^{1}H NMR spectrum of the compound **2i**, DMSO-*d*6.

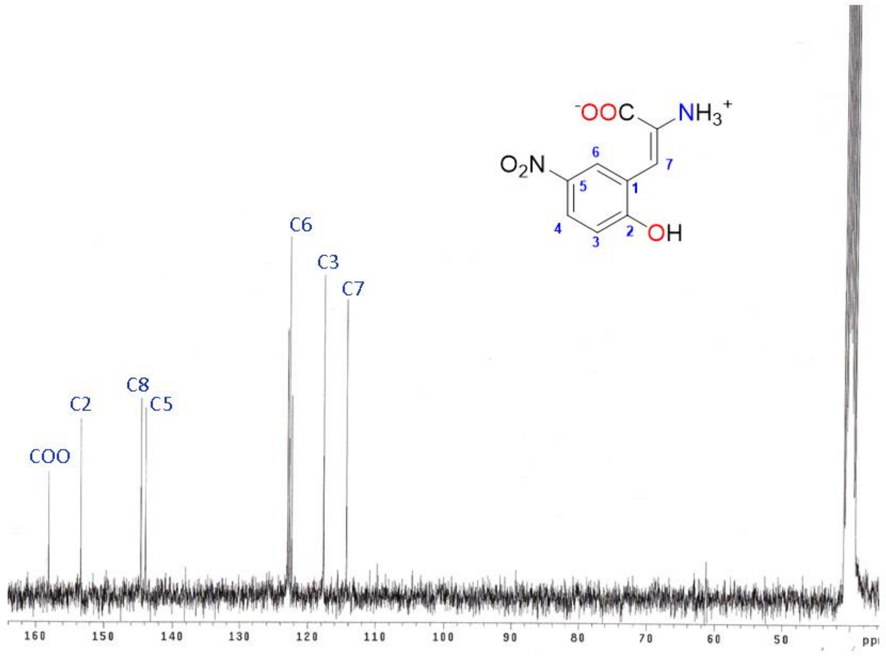

Figure S34. RMN ^{13}C NMR spectrum of the compound **2i**, DMSO-*d*6.

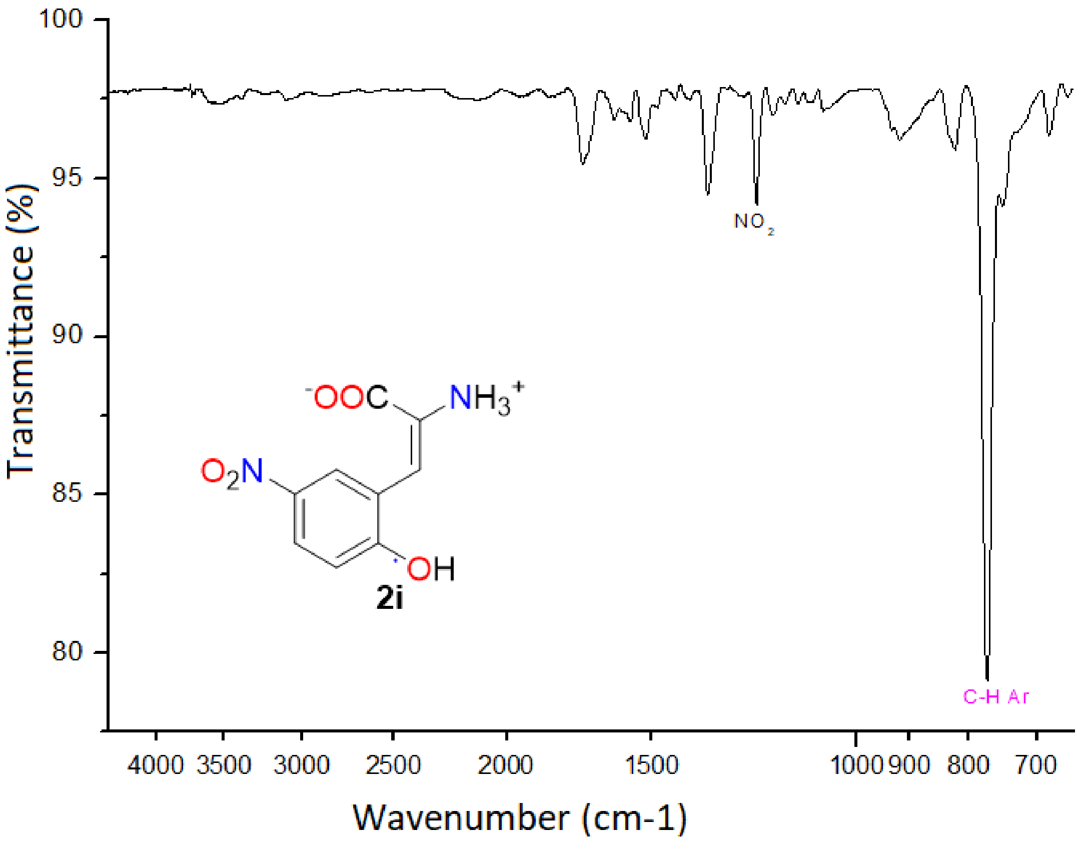

Figure S35. IR spectrum of compound **2i**.

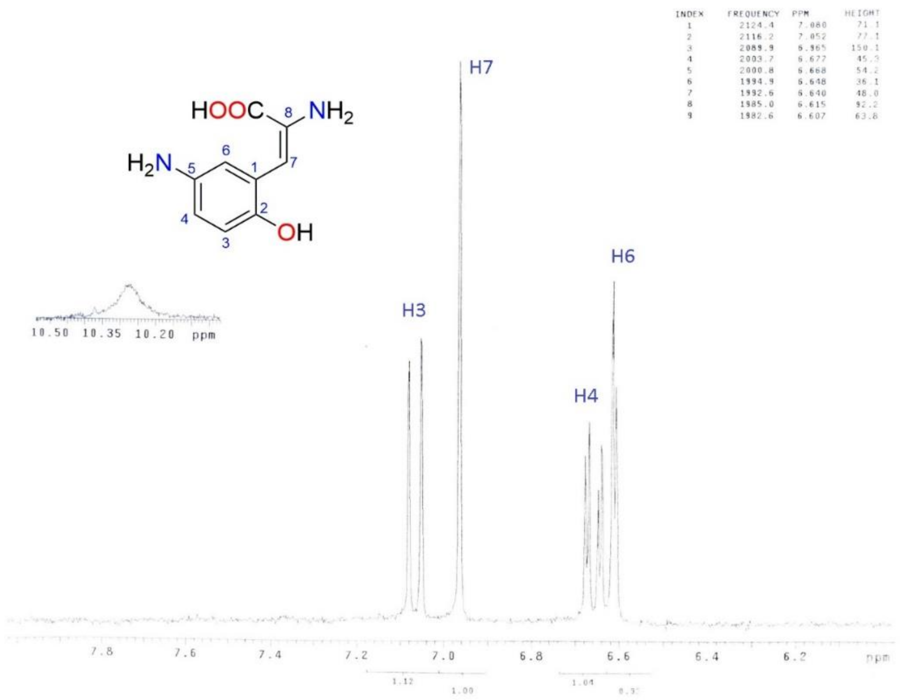

Figure S36. ^{1}H NMR spectrum of compound **2j**, DMSO-*d*6.

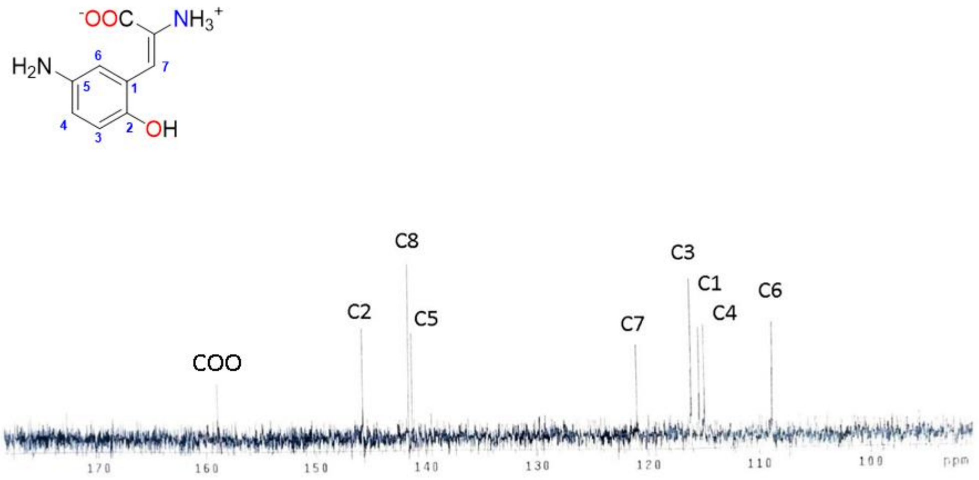

Figure S37. ^{13}C NMR spectrum of compound **2j**, DMSO-*d6*.

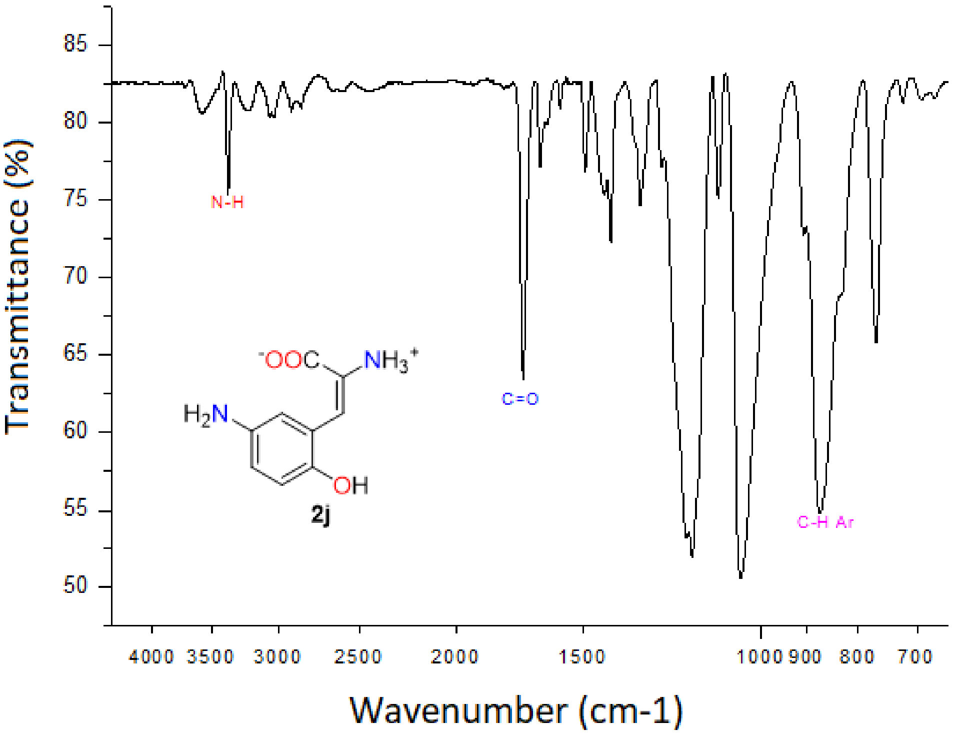

Figure S38. IR spectrum of compound **2j**.

7. MO calculations at B3LYP/6-31+G(d,p). Energies and imaginary frequencies of **2a-j**.

Compound	Energy in Hartrees				Imaginary frequencies			
	Neutral	Radical		Cation radical	Neutral	Radical		Cation radical
**2a**	-628.788495931	-628.1788919	-	-628.568361494	0	0	-	0
**2b**	-3199.91363217	-3199.318232	-	-3199.68920784	0	0	-	0
**2c**	-1088.37965205	-1087.748126	-	-1088.15219376	0	0	-	0
**2d**	-728.026669500	-727.4171962	-	-727.795798217	0	0	-	0
**2e**	-782.634891372	-782.0238055	-	-782.421421711	0	0	-	0
**2f**	-704.014229880	-703.3974001	-703.4155184	-703.799487241	0	0	0	0
**2g**	-743.352620614	-742.7258151	-	-743.103712332	0	0	-	0
**2h**	-704.009648209	-703.3989183	-703.4130263	-703.793422537	0	0	0	0
**2i**	-833.298569285	-832.6616767	-	-833.056312738	0	0	-	0
**2j**	-684.182205499	-683.5391383	-	-683.943797537	0	0	-	0

9. Z-matrix of **2a-j** in neutral, radical and cation radical forms.

R(U)B3LYP/6-31+G(d,p) Z- Matrix

2a

Neutral				Radical				Cation radical			
C	0.703717	0.234081	-0.286643	C	0.822393	0.693841	-0.022511	C	0.836403	0.679248	-0.055827
C	1.845096	0.893783	0.22206	C	0.997423	-0.705427	-0.475233	C	1.06545	-0.676497	-0.483159
C	0.873175	-1.088392	-0.733975	C	1.976382	1.4273	0.349322	C	1.974092	1.435533	0.367831
C	3.078891	0.244425	0.313535	C	2.335837	-1.267956	-0.399235	C	2.338297	-1.242539	-0.396233
C	2.09873	-1.74576	-0.644886	C	3.231246	0.848544	0.377235	C	3.223949	0.866531	0.456145
C	3.206223	-1.077454	-0.115109	C	3.406345	-0.517134	0.005707	C	3.403717	-0.483674	0.077589
C	-0.543219	0.992497	-0.423986	C	-0.440138	1.351924	-0.07274	C	-0.416514	1.336014	-0.126811
O	1.682163	2.19481	0.629456	O	0.046727	-1.369645	-0.950925	O	0.018806	-1.330026	-1.024664
C	-1.84067	0.617857	-0.268027	C	-1.711031	0.815504	-0.10896	C	-1.718362	0.803154	-0.155455
N	-2.900104	1.470901	-0.583729	N	-2.803823	1.559713	-0.461632	N	-2.749743	1.511462	-0.612774
C	-2.344318	-0.650894	0.340548	C	-2.120982	-0.55732	0.347491	C	-2.174305	-0.519297	0.442535
O	-3.50778	-1.00853	0.240813	O	-3.125678	-1.10403	-0.067797	O	-3.227804	-1.018697	0.121954
O	-1.445469	-1.343225	1.068362	O	-1.384246	-1.032069	1.365363	O	-1.362602	-0.9567	1.40628
H	-0.399315	2.029317	-0.726716	H	-0.394148	2.438374	-0.1566	H	-0.367364	2.423437	-0.173916
H	0.024496	-1.598474	-1.176728	H	1.858453	2.46721	0.644537	H	1.823641	2.470893	0.657762
H	3.936754	0.774335	0.722774	H	2.441221	-2.303724	-0.706085	H	4.067977	1.446912	0.811579
H	2.19364	-2.766468	-1.002079	H	4.09021	1.433703	0.691462	H	4.389794	-0.933335	0.138526
H	2.529583	2.553087	0.923948	H	4.400206	-0.955138	0.041032	H	-2.631023	2.409034	-1.065921
H	-2.735434	2.130383	-1.329923	H	-2.675183	2.391204	-1.018129	H	-3.677044	1.094792	-0.592139
H	-3.800487	1.011328	-0.629825	H	-3.668398	1.051372	-0.595826	H	-1.749136	-1.763421	1.793305
H	-1.914141	-2.119638	1.418367	H	-1.704874	-1.930696	1.551446	H	2.496125	-2.267714	-0.719957
H	4.168583	-1.575467	-0.043852					H	0.271663	-2.208278	-1.348067

2b

Neutral				Radical				Cation radical			
C	-0.612742	0.997933	-0.236532	C	-0.397496	-0.286159	-0.263807	C	-0.389188	-0.213801	-0.396047
C	-0.174263	2.283479	0.152911	C	-0.642585	1.156145	-0.482785	C	-0.549798	1.210729	-0.546112
C	0.357213	-0.01117	-0.358904	C	0.932391	-0.729601	-0.097639	C	0.930629	-0.713169	-0.208721
C	1.16875	2.527804	0.445704	C	0.501086	2.046212	-0.382907	C	0.543154	2.069048	-0.373232
C	1.689697	0.237625	-0.05212	C	1.979236	0.17266	-0.058481	C	1.996669	0.145206	-0.058955
C	2.110908	1.503575	0.350671	C	1.77041	1.573426	-0.192344	C	1.802507	1.549221	-0.129714
C	-2.024088	0.810558	-0.580821	C	-1.452372	-1.24613	-0.328523	C	-1.449308	-1.152922	-0.505295
O	-1.12639	3.266509	0.239052	O	-1.775248	1.596109	-0.787154	O	-1.755686	1.655333	-0.928277
C	-2.859231	-0.244265	-0.384342	C	-2.800538	-1.052154	-0.141596	C	-2.81411	-1.010803	-0.22548
N	-4.149704	-0.273205	-0.906781	N	-3.72521	-2.018553	-0.44129	N	-3.701504	-1.926968	-0.631326
C	-2.606458	-1.446817	0.471271	C	-3.380988	0.128446	0.592814	C	-3.360527	0.049485	0.712535
O	-3.297583	-2.450254	0.40687	O	-2.924226	0.601729	1.606786	O	-2.688897	0.639788	1.5199
O	-1.60613	-1.320785	1.364492	O	-4.579319	0.493942	0.070591	O	-4.694508	0.16552	0.576982
H	-2.468066	1.658566	-1.100919	H	-1.153475	-2.259798	-0.595592	H	-1.149206	-2.152727	-0.815895
H	0.061194	-0.991847	-0.709078	H	1.129047	-1.790148	0.023019	H	1.080097	-1.785035	-0.14024
H	1.483472	3.521597	0.7564	H	0.302996	3.106368	-0.502452	H	0.406898	3.143115	-0.463094

H	-0.712111	4.10581	0.478199	H	2.617886	2.24875	-0.143034	H	2.649984	2.21523	-0.009383
H	-4.304895	0.242198	-1.760415	H	-3.464671	-2.770278	-1.062375	H	-3.436265	-2.689592	-1.242521
H	-4.59282	-1.182349	-0.878696	H	-4.701865	-1.765033	-0.430916	H	-4.676384	-1.846336	-0.367667
H	-1.563033	-2.158885	1.85557	H	-4.907307	1.23601	0.606025	H	-5.030101	0.785513	1.249402
H	3.15282	1.694968	0.580099	Br	3.755124	-0.45678	0.177262	Br	3.733974	-0.516744	0.23591
Br	2.969527	-1.168392	-0.222973					H	-1.771004	2.621571	-1.010877

2c

Neutral				Radical				Cation radical			
C	0.095521	0.794513	-0.240335	C	0.223509	-0.387361	-0.220388	C	0.230315	-0.333534	-0.332017
C	0.865243	1.904211	0.175599	C	0.079602	1.063341	-0.474372	C	0.178792	1.092783	-0.537122
C	0.765807	-0.426746	-0.426334	C	1.51864	-0.911716	-0.020091	C	1.507019	-0.919022	-0.094892
C	2.231917	1.778169	0.433112	C	1.279958	1.877442	-0.380758	C	1.329314	1.874887	-0.37436
C	2.125944	-0.544673	-0.161347	C	2.62344	-0.079973	0.021966	C	2.62909	-0.134488	0.054159
C	2.873303	0.549807	0.271398	C	2.511081	1.328235	-0.148123	C	2.540726	1.276566	-0.072985
C	-1.320362	0.999484	-0.559361	C	-0.889744	-1.27976	-0.288301	C	-0.889597	-1.200433	-0.435638
O	0.206491	3.098238	0.323359	O	-1.016505	1.57115	-0.80581	O	-0.982952	1.608781	-0.964277
C	-2.40424	0.201138	-0.368233	C	-2.225928	-0.996031	-0.133276	C	-2.249977	-0.951606	-0.208242
N	-3.664053	0.533393	-0.859867	N	-3.204959	-1.906742	-0.435282	N	-3.185919	-1.81781	-0.612726
C	-2.468794	-1.049971	0.451399	C	-2.744623	0.233734	0.565671	C	-2.750306	0.178956	0.671861
O	-3.409678	-1.824231	0.387686	O	-2.278246	0.699313	1.578811	O	-2.064733	0.749645	1.481344
O	-1.44899	-1.230176	1.312803	O	-3.907568	0.662466	0.012194	O	-4.067272	0.382642	0.484705
H	-1.529216	1.950862	-1.047154	H	-0.651368	-2.315165	-0.531422	H	-0.652147	-2.230675	-0.696911
H	0.218428	-1.284381	-0.796941	H	1.646801	-1.978978	0.131163	H	1.579997	-1.994924	0.020123
H	2.801662	2.64251	0.766901	H	1.154329	2.944686	-0.530647	H	1.274688	2.951333	-0.509698
H	0.831375	3.789809	0.577139	H	3.402746	1.943891	-0.095051	H	3.433586	1.880474	0.047772
H	-3.690618	1.095725	-1.697377	H	-2.981024	-2.685859	-1.036505	H	-2.955632	-2.620497	-1.185792
H	-4.334302	-0.22426	-0.84107	H	-4.163326	-1.591383	-0.449885	H	-4.160978	-1.658928	-0.387467
H	-1.626938	-2.061122	1.785329	H	-4.19825	1.434526	0.526403	H	-4.380372	1.049691	1.12207
H	3.934227	0.45005	0.47058	Cl	4.211044	-0.762084	0.306906	Cl	4.167967	-0.850242	0.407473
Cl	2.922067	-2.098776	-0.402618					H	-0.925736	2.569127	-1.086814

2d

Neutral				Radical				Cation radical			
C	0.458306	0.524435	-0.241243	C	0.532669	0.509621	-0.143473	C	0.549355	0.494098	-0.194036
C	1.462245	1.416011	0.203575	C	0.544129	-0.924058	-0.515163	C	0.611587	-0.907533	-0.525953
C	0.843342	-0.799116	-0.519119	C	1.76307	1.1376	0.151157	C	1.772346	1.152875	0.124758
C	2.777192	0.987906	0.397803	C	1.818987	-1.622526	-0.456318	C	1.821099	-1.608422	-0.446266
C	2.151987	-1.20281	-0.31392	C	2.929483	0.404456	0.160233	C	2.937735	0.43328	0.196278
C	3.137008	-0.337113	0.141973	C	2.983601	-0.978506	-0.132966	C	2.984662	-0.94972	-0.078603
C	-0.885346	1.052125	-0.491054	C	-0.657109	1.298239	-0.202027	C	-0.632773	1.27458	-0.274958
O	1.078822	2.713339	0.444522	O	-0.48857	-1.514158	-0.911046	O	-0.521352	-1.477094	-0.977233
C	-2.114931	0.495669	-0.323748	C	-1.9746	0.901924	-0.141438	C	-1.979633	0.882009	-0.192903
N	-3.278224	1.134694	-0.746759	N	-3.00274	1.73287	-0.493353	N	-2.959748	1.655864	-0.654952
C	-2.442203	-0.771763	0.401769	C	-2.500019	-0.387644	0.429251	C	-2.531618	-0.333837	0.538768
O	-3.532976	-1.311801	0.313455	O	-3.575236	-0.850683	0.099307	O	-3.648247	-0.740535	0.317748
O	-1.474038	-1.241549	1.212303	O	-1.761133	-0.872216	1.440791	O	-1.710446	-0.780416	1.490508
H	-0.890803	2.063289	-0.896035	H	-0.499535	2.362204	-0.380095	H	-0.474508	2.341411	-0.426856
H	0.127114	-1.510417	-0.91127	H	1.80025	2.193521	0.400338	H	1.779678	2.213157	0.352408
H	3.526996	1.690321	0.754411	H	1.803004	-2.678178	-0.706045	H	1.850727	-2.66587	-0.692879
H	1.846435	3.239932	0.701986	H	3.940079	-1.489983	-0.099445	H	3.932858	-1.472921	-0.013718

2e

Neutral				Radical				Cation radical			
H	-3.192953	1.752585	-1.540051	H	-2.819649	2.512451	-1.106709	H	-2.779	2.497118	-1.188731
H	-4.098743	0.542983	-0.761949	H	-3.921342	1.312886	-0.551452	H	-3.921726	1.343889	-0.548693
H	-1.826808	-2.045838	1.629526	H	-2.166408	-1.714099	1.709464	H	-2.154911	-1.505576	1.966818
H	4.153161	-0.687603	0.283503	F	4.093447	1.02588	0.473272	F	4.079323	1.046607	0.538024
F	2.489235	-2.495565	-0.592363	C	0.597293	1.311164	-0.087598	H	-0.38498	-2.401345	-1.236597
C	0.451343	-0.027704	-0.465672					C	0.571395	1.324856	-0.114777
C	-0.807333	-0.57276	-0.162728	C	-0.296965	0.153769	-0.28707	C	-0.342218	0.230185	-0.249505
C	0.505918	1.343502	-0.795254	C	0.028223	2.564709	0.20415	C	0.019271	2.611641	0.181985
C	-1.965193	0.22928	-0.153035	C	-1.740247	0.371537	-0.055716	C	-1.729101	0.404818	-0.019274
C	-0.634603	2.134793	-0.783516	C	-1.340736	2.71556	0.384841	C	-1.325957	2.762892	0.412669
C	-1.884547	1.585098	-0.457347	C	-2.231093	1.622243	0.267139	C	-2.213888	1.658763	0.317521
C	1.597877	-0.938533	-0.534203	C	2.010784	1.212756	-0.316284	C	1.962429	1.218849	-0.362455
O	-0.908128	-1.902621	0.134659	O	0.119216	-0.956401	-0.673973	O	0.103297	-0.960226	-0.66749
C	2.905432	-0.761031	-0.207882	C	2.842766	0.122456	-0.260329	C	2.805518	0.095888	-0.322295
N	3.875079	-1.728326	-0.483312	N	4.127889	0.153476	-0.750596	N	3.988635	0.090944	-0.938918
C	3.494961	0.377683	0.558863	C	2.565723	-1.181907	0.431138	C	2.609522	-1.163262	0.507218
O	4.698197	0.583106	0.606767	O	3.123342	-2.216731	0.114674	O	3.219205	-2.177566	0.257258
O	2.616078	1.125525	1.255163	O	1.781137	-1.068841	1.516501	O	1.820623	-0.968971	1.565453
H	1.3569	-1.920242	-0.941347	H	2.486017	2.140675	-0.636314	H	2.454694	2.151515	-0.635797
H	1.457882	1.776276	-1.080358	H	0.681023	3.426119	0.316394	H	0.690558	3.459983	0.261765
O	-3.111071	-0.461064	0.173749	O	-2.481447	-0.743072	-0.208199	O	-2.441048	-0.736628	-0.194644
H	-0.567958	3.186387	-1.044624	H	-1.743786	3.692762	0.634069	H	-1.728122	3.736353	0.671154
H	-2.769423	2.210451	-0.456302	H	-3.290073	1.783866	0.429927	H	-3.272425	1.811262	0.491126
H	-1.843712	-2.097252	0.301736	H	4.346781	0.819326	-1.476442	H	4.277519	0.837607	-1.558109
H	3.712892	-2.301436	-1.298332	H	4.587993	-0.744922	-0.823613	H	4.574561	-0.736243	-0.866031
H	4.826068	-1.392631	-0.399239	H	1.632459	-1.96935	1.850897	H	1.790802	-1.795118	2.081351
H	3.137369	1.804018	1.716475	C	-3.902192	-0.662085	-0.041334	C	-3.885452	-0.710884	-0.038543
C	-4.354994	0.246996	0.216586	C	-4.47281	-2.048635	-0.278259	C	-4.406415	-2.108217	-0.302724
C	-5.441828	-0.738521	0.606081	H	-4.1297	-0.307375	0.973335	H	-4.115029	-0.381607	0.981523
H	-4.281848	1.062461	0.9486	H	-4.3103	0.062975	-0.758689	H	-4.294983	0.014426	-0.751298
H	-4.555281	0.686125	-0.769994	H	-4.236067	-2.396451	-1.287709	H	-4.166869	-2.432246	-1.319381
H	-5.5134	-1.549378	-0.125162	H	-4.060592	-2.764477	0.438609	H	-3.987283	-2.825579	0.408357
H	-5.23931	-1.172581	1.589829	H	-5.561443	-2.026523	-0.162393	H	-5.494768	-2.114139	-0.191065
H	-6.408553	-0.226314	0.647852					H	-0.656335	-1.568874	-0.744711

2f

Neutral

C	0.304604	0.41177	-0.275323
C	1.362087	1.208412	0.223681
C	0.643907	-0.879047	-0.712647
C	2.667205	0.725535	0.319038
C	1.939701	-1.382188	-0.626595
C	2.954388	-0.573858	-0.102178
C	-1.025404	1.008077	-0.412479
O	1.04526	2.480547	0.622708
C	-2.270259	0.478245	-0.269899
N	-3.419908	1.207503	-0.593665
C	-2.627165	-0.838582	0.333148
O	-3.753342	-1.308069	0.264484
O	-1.644528	-1.454081	1.024668
H	-1.010696	2.058088	-0.703855
H	-0.12947	-1.500619	-1.149348
H	3.466502	1.34221	0.72081
H	2.162779	-2.384972	-0.982322
O	4.253559	-0.992674	0.013185
H	1.842954	2.942097	0.912485
H	-3.330092	1.8585	-1.359918
H	-4.261914	0.647264	-0.630685
H	-2.035584	-2.267266	1.386048
H	4.332909	-1.903775	-0.298386

Radical (C2)

C	-0.389143	0.812987	-0.022124
C	-0.65785	-0.573376	-0.4904
C	-1.496433	1.623646	0.344635
C	-2.022038	-1.046629	-0.426351
C	-2.782049	1.134261	0.359768
C	-3.038268	-0.224205	-0.021214
C	0.909749	1.377182	-0.05122
O	0.258538	-1.285433	-0.960336
C	2.139004	0.74017	-0.103753
N	3.277388	1.399158	-0.473805
C	2.446543	-0.656474	0.356011
O	3.412155	-1.273459	-0.05467
O	1.678204	-1.072016	1.376721
H	0.95051	2.466147	-0.100838
H	-1.315064	2.652213	0.645865
H	-2.220495	-2.065255	-0.739358
H	-3.608581	1.771149	0.66641
O	-4.313324	-0.716334	0.004859
H	3.207152	2.235781	-1.032928
H	4.104506	0.830135	-0.602153
H	1.927243	-1.993207	1.561363
H	-4.928677	-0.038513	0.313574

Radical (C4)

C	-0.447575	0.76799	-0.090384
C	-0.800929	-0.568327	-0.515563
C	-1.530728	1.616095	0.323618
C	-2.086579	-1.028505	-0.431577
C	-2.818308	1.176628	0.424927
C	-3.172103	-0.196532	0.065781
C	0.856944	1.330688	-0.153896
O	0.203811	-1.315365	-1.069021
C	2.109081	0.740025	-0.127707
N	3.237578	1.42813	-0.492892
C	2.448988	-0.601429	0.437922
O	3.496436	-1.172103	0.184551
O	1.564659	-1.054892	1.342402
H	0.87387	2.416502	-0.256948
H	-1.284768	2.638382	0.600117
H	-2.349571	-2.027976	-0.769351
H	-3.61905	1.820583	0.773389
O	-4.342145	-0.621026	0.155991
H	-0.158864	-2.147536	-1.401602
H	3.157003	2.21412	-1.119324
H	4.097263	0.89658	-0.532566
H	1.886236	-1.920284	1.646409

Cation radical

C	-0.40543	0.79017	-0.072338
C	-0.738851	-0.544637	-0.508803
C	-1.493467	1.624138	0.338891
C	-2.038497	-1.015164	-0.440157
C	-2.782622	1.16214	0.416788
C	-3.059647	-0.177564	0.034801
C	0.889527	1.354568	-0.121571
O	0.262671	-1.272064	-1.04297
C	2.149357	0.734047	-0.138001
N	3.239182	1.378102	-0.565382
C	2.501108	-0.622353	0.445171
O	3.524488	-1.191038	0.139748
O	1.643268	-1.021594	1.387906
H	0.918566	2.443108	-0.159005
H	-1.274233	2.64604	0.631938
H	-2.295939	-2.016127	-0.772234
H	-3.58384	1.807291	0.764325
O	-4.288067	-0.701396	0.08113
H	3.189264	2.2838	-1.013367
H	4.134137	0.898397	-0.534816
H	1.969528	-1.860264	1.761618
H	-4.947252	-0.068558	0.405428
H	-0.052248	-2.123725	-1.381413

2g

Neutral				Radical				Cation radical			
C	0.200935	0.822583	-0.234917	C	0.244645	-0.257965	-0.254292	C	0.255607	-0.249067	-0.310297
C	0.992683	1.928814	0.12901	C	0.033498	1.184517	-0.493088	C	0.102656	1.174022	-0.499466
C	0.834384	-0.43118	-0.361092	C	1.557651	-0.729354	-0.043784	C	1.56521	-0.747528	-0.090651
C	2.356299	1.772587	0.395297	C	1.203526	2.044388	-0.409831	C	1.202825	2.037385	-0.367482
C	2.194133	-0.586814	-0.094372	C	2.640283	0.143704	0.008324	C	2.646289	0.113462	0.034577
C	2.958941	0.523707	0.289396	C	2.452174	1.547421	-0.172548	C	2.452787	1.52433	-0.098577
C	-1.205928	1.052524	-0.575332	C	-0.826385	-1.205897	-0.363132	C	-0.817117	-1.175291	-0.458149
O	0.367603	3.152179	0.221252	O	-1.088631	1.655614	-0.795658	O	-1.107759	1.616405	-0.877942
C	-2.317294	0.295594	-0.372577	C	-2.177837	-1.021278	-0.207127	C	-2.1864	-1.001042	-0.258478
N	-3.557826	0.647544	-0.909669	N	-3.099206	-1.963985	-0.59815	N	-3.087343	-1.871421	-0.730508
C	-2.441732	-0.907254	0.504436	C	-2.847958	0.116788	0.50959	C	-2.850389	0.052106	0.612569
O	-3.409355	-1.651722	0.463144	O	-3.995426	0.447357	0.274057	O	-4.025726	0.315203	0.499436
O	-1.448339	-1.081629	1.399912	O	-2.131357	0.61337	1.532877	O	-2.039498	0.538028	1.558665
H	-1.38213	1.989284	-1.10337	H	-0.527976	-2.206986	-0.676697	H	-0.521632	-2.174038	-0.775676
H	0.238415	-1.272012	-0.689604	H	1.704064	-1.793456	0.105231	H	1.69311	-1.816851	0.022542
H	2.951351	2.634514	0.689877	H	1.034632	3.105117	-0.564802	H	1.068191	3.107433	-0.499003
O	2.877715	-1.773619	-0.197239	O	3.927216	-0.226883	0.235713	O	3.910778	-0.246358	0.288684
H	4.017411	0.393201	0.487948	H	3.325617	2.19	-0.119092	H	3.317543	2.171535	0.002692
H	1.018359	3.834344	0.430126	H	-2.842776	-2.610135	-1.329486	H	-2.831395	-2.619268	-1.360928
H	-3.539584	1.145054	-1.787717	H	-4.063113	-1.655538	-0.600723	H	-4.070797	-1.713852	-0.536175
H	-4.250244	-0.089129	-0.867202	H	-2.635369	1.363547	1.89071	H	-2.556616	1.148569	2.114804
H	-1.684253	-1.868522	1.919734	C	4.22641	-1.606566	0.433709	C	4.242809	-1.636504	0.436147
C	2.166388	-2.927962	-0.61832	H	3.704759	-2.000853	1.313662	H	3.706677	-2.070493	1.286353
H	1.355733	-3.178015	0.078479	H	3.960684	-2.19919	-0.449672	H	4.014455	-2.18544	-0.483209
H	1.750978	-2.798885	-1.626357	H	5.303461	-1.654829	0.595086	H	5.314679	-1.658631	0.624092
H	2.894887	-3.739908	-0.629955					H	-1.110833	2.573526	-1.03384

2h

Neutral

C	0.451928	0.538471	-0.244353
C	1.445352	1.44173	0.19918
C	0.843849	-0.780723	-0.518945
C	2.758119	1.017308	0.394285
C	2.154754	-1.208235	-0.31638
C	3.120067	-0.309253	0.142358
C	-0.897282	1.052741	-0.496637
O	1.052125	2.74002	0.43671
C	-2.119484	0.482941	-0.325458
N	-3.292589	1.103612	-0.756074
C	-2.429375	-0.783315	0.408289
O	-3.504291	-1.352964	0.304197
O	-1.468605	-1.216512	1.246963
H	-0.913947	2.059847	-0.911743
H	0.125176	-1.489896	-0.911913
H	3.507953	1.720661	0.74976
O	2.43759	-2.525567	-0.604159
H	4.147637	-0.629572	0.297163
H	1.817687	3.270681	0.691107
H	-3.210604	1.70605	-1.561815
H	-4.099538	0.493279	-0.776219
H	-1.806283	-2.029517	1.659393
H	3.377919	-2.696629	-0.46693

Radical (C2)

C	-0.524094	0.498404	-0.160037
C	-0.537989	-0.934876	-0.525255
C	-1.741708	1.127179	0.140173
C	-1.816348	-1.621735	-0.460912
C	-2.940594	0.419685	0.165162
C	-2.975689	-0.969732	-0.1319
C	0.67331	1.289808	-0.237125
O	0.490151	-1.537192	-0.918088
C	1.985725	0.904387	-0.144438
N	3.024465	1.73958	-0.48077
C	2.502079	-0.380021	0.442034
O	3.580983	-0.84924	0.131447
O	1.747349	-0.862433	1.444274
H	0.512522	2.344545	-0.461356
H	-1.764879	2.184718	0.385997
H	-1.810246	-2.678053	-0.709047
O	-4.071617	1.108066	0.491395
H	-3.923987	-1.5033	-0.100937
H	2.845801	2.489981	-1.131152
H	3.934085	1.301699	-0.549257
H	2.148216	-1.704921	1.717208
H	-4.835852	0.516079	0.495513

Radical (C5)

C	0.502366	0.472809	-0.277429
C	1.526829	1.347521	0.196872
C	0.862718	-0.835487	-0.551127
C	2.846454	0.898759	0.422511
C	2.200797	-1.345551	-0.33444
C	3.187784	-0.409991	0.17443
C	-0.827173	1.041071	-0.53811
O	1.171574	2.638875	0.430678
C	-2.061895	0.524368	-0.311533
N	-3.224536	1.199869	-0.680952
C	-2.298612	-0.73312	0.469707
O	-1.518489	-1.279157	1.217539
O	-3.56167	-1.211803	0.274625
H	-0.811202	2.034685	-0.983901
H	0.140648	-1.536244	-0.953281
H	3.586712	1.602623	0.799267
O	2.50108	-2.542001	-0.592914
H	4.194137	-0.779562	0.34129
H	1.935352	3.146211	0.738701
H	-3.131276	1.878725	-1.422786
H	-4.065325	0.644153	-0.734286
H	-3.652856	-1.999261	0.836938

Cation radical

C	-0.539952	0.491804	-0.206638
C	-0.599657	-0.91488	-0.531612
C	-1.755213	1.144692	0.107054
C	-1.809461	-1.615553	-0.436874
C	-2.946489	0.444755	0.195105
C	-2.968605	-0.953566	-0.072645
C	0.647949	1.270333	-0.30566
O	0.525552	-1.485448	-0.996402
C	1.987289	0.885632	-0.192498
N	2.981389	1.664528	-0.628625
C	2.526031	-0.330354	0.544352
O	3.646461	-0.740202	0.346093
O	1.689464	-0.784728	1.482034
H	0.487872	2.329934	-0.498703
H	-1.756522	2.206532	0.327712
H	-1.840491	-2.67558	-0.672433
O	-4.054231	1.129847	0.542864
H	-3.903843	-1.503134	-0.006805
H	2.810367	2.503814	-1.166975
H	3.940131	1.355188	-0.500674
H	2.132233	-1.510368	1.958616
H	-4.836189	0.562245	0.601368
H	0.387403	-2.41326	-1.241947

2i

Neutral				Radical				Cation radical			
C	-0.057964	-0.129589	-0.515652	C	0.004821	0.346161	-0.226613	C	0.000187	-0.26431	-0.360395
C	-0.043062	1.284986	-0.598483	C	0.198917	-1.107745	-0.463528	C	-0.09074	1.162693	-0.536736
C	1.155071	-0.769563	-0.239263	C	-1.310946	0.834973	-0.05954	C	1.296154	-0.820067	-0.151052
C	1.13361	2.010339	-0.364598	C	-0.97715	-1.961012	-0.363995	C	1.039891	1.972958	-0.379152
C	2.319074	-0.035218	-0.011846	C	-2.380212	-0.035211	-0.01893	C	2.384495	0.004306	-0.003959
C	2.323548	1.358662	-0.065377	C	-2.22285	-1.44741	-0.157954	C	2.274365	1.405202	-0.10284
C	-1.245091	-0.944972	-0.827281	C	1.085009	1.263471	-0.272143	C	-1.090332	-1.167075	-0.450265
O	-1.202162	1.91153	-0.934238	O	1.312022	-1.572195	-0.783068	O	-1.276209	1.652585	-0.924078
C	-2.51796	-0.861036	-0.359971	C	2.438317	1.002618	-0.137517	C	-2.463249	-0.962405	-0.214085
N	-3.567281	-1.580285	-0.901213	N	3.380756	1.923464	-0.47163	N	-3.365878	-1.851033	-0.631424
C	-2.975499	-0.033604	0.801705	C	2.993581	-0.208381	0.570632	C	-2.995008	0.136308	0.689959
O	-4.14596	0.041155	1.132383	O	2.543592	-0.662009	1.59554	O	-2.31818	0.709034	1.504164
O	-1.990273	0.553825	1.507429	O	4.160668	-0.611328	0.013046	O	-4.317125	0.298456	0.511879
H	-1.067395	-1.718535	-1.57508	H	0.821519	2.301494	-0.4734	H	-0.821137	-2.190372	-0.708836
H	1.197402	-1.850954	-0.188547	H	-1.492067	1.896642	0.064746	H	1.426164	-1.892839	-0.061188
H	1.115378	3.095678	-0.429691	H	-0.814801	-3.025777	-0.49207	H	0.952297	3.049233	-0.496258
H	3.24003	1.906015	0.115209	H	-3.101646	-2.078087	-0.102425	H	3.16121	2.016878	0.018256
H	-1.05896	2.86507	-1.001004	H	3.129935	2.739785	-1.009661	H	-3.109332	-2.642878	-1.210356
H	-3.354548	-2.339837	-1.529355	H	4.358178	1.677355	-0.424184	H	-4.347411	-1.724958	-0.407632
H	-4.393618	-1.681131	-0.329177	H	4.480393	-1.369027	0.531925	H	-4.654397	0.941776	1.161756
H	-2.420195	1.023725	2.24178	N	-3.723013	0.506445	0.180047	N	3.711484	-0.595869	0.257441
N	3.564528	-0.743623	0.285446	O	-3.84904	1.730747	0.289762	O	3.767019	-1.820008	0.331511
O	3.53921	-1.977572	0.305424	O	-4.662571	-0.294781	0.225179	O	4.655511	0.178715	0.377941
O	4.575536	-0.068025	0.500355					H	-1.249342	2.615551	-1.038036

2j

Neutral				Radical				Cation radical			
C	0.456914	0.522862	-0.244456	C	-0.51713	0.480717	-0.184375	C	-0.520155	0.489411	-0.236815
C	1.381501	1.477993	0.233268	C	-0.524831	-0.954608	-0.536586	C	-0.595648	-0.921044	-0.550633
C	0.933132	-0.769221	-0.518498	C	-1.73073	1.103882	0.119973	C	-1.719882	1.138214	0.086751
C	2.71045	1.124216	0.458842	C	-1.80566	-1.636626	-0.473196	C	-1.810203	-1.623625	-0.431422
C	2.264508	-1.138754	-0.294739	C	-2.947751	0.405097	0.160149	C	-2.9354	0.447133	0.195405
C	3.153734	-0.173523	0.200394	C	-2.964859	-0.989083	-0.143471	C	-2.959853	-0.964467	-0.063045
C	-0.905765	0.972874	-0.54369	C	0.681801	1.279344	-0.279875	C	0.683957	1.258432	-0.381961
O	0.908887	2.751561	0.474927	O	0.50713	-1.56212	-0.911006	O	0.512631	-1.504256	-1.029011
C	-2.129003	0.393593	-0.395612	C	1.991271	0.907565	-0.155591	C	2.004607	0.88389	-0.204037
N	-3.276104	0.954668	-0.961025	N	3.036691	1.741993	-0.497593	N	3.032362	1.650627	-0.611102
C	-2.476282	-0.814147	0.407604	C	2.500454	-0.360159	0.467515	C	2.503999	-0.320372	0.570649
O	-3.633196	-1.152176	0.607336	O	3.586609	-0.833954	0.188191	O	3.631376	-0.740959	0.439446
O	-1.442593	-1.483942	0.961241	O	1.727943	-0.828158	1.464124	O	1.622006	-0.777277	1.471195
H	-0.935147	1.972046	-0.979106	H	0.517506	2.321505	-0.556062	H	0.528818	2.295113	-0.67516
H	0.245343	-1.497705	-0.93332	H	-1.731257	2.166404	0.355897	H	-1.694524	2.202911	0.297662
H	3.409464	1.864499	0.842777	H	-1.799723	-2.693839	-0.71862	H	-1.839967	-2.686701	-0.653941
N	2.716529	-2.427387	-0.638579	N	-4.129852	1.054169	0.451534	N	-4.084568	1.0723	0.549598
H	4.195952	-0.429605	0.372331	H	-3.910232	-1.526433	-0.116475	H	-3.895654	-1.508826	0.020049
H	1.642907	3.324176	0.731297	H	2.861389	2.445596	-1.199753	H	2.891733	2.466038	-1.189989
H	-3.203721	1.921051	-1.246283	H	3.938701	1.287227	-0.563073	H	3.979241	1.333137	-0.440876
H	-4.140811	0.715467	-0.49281	H	2.12351	-1.667705	1.753068	H	2.050825	-1.49954	1.964549
H	-1.841214	-2.185731	1.503279	H	-4.930513	0.50069	0.717651	H	-4.949957	0.564529	0.657431
H	3.543489	-2.731748	-0.141587	H	-4.086105	1.9705	0.87205	H	-4.105886	2.062262	0.746086
H	2.000844	-3.142318	-0.626062					H	0.36416	-2.434907	-1.257666
